# Dietary Salt Intake and Gastric Cancer Risk: A Systematic Review and Meta-Analysis

**DOI:** 10.3389/fnut.2021.801228

**Published:** 2021-12-08

**Authors:** Bo Wu, Dehua Yang, Shuhan Yang, Guangzhe Zhang

**Affiliations:** Department of Anorectal Surgery, The First Hospital of China Medical University, Shenyang, China

**Keywords:** gastric cancer, salt intake, risks, meta-analysis, systematic review

## Abstract

The results of prospective cohort studies regarding the role of salt intake and subsequent gastric cancer risk are inconsistent. Thus, we performed a systematic review and meta-analysis to summarize the strength of the association of salt intake with gastric cancer morbidity and mortality. PubMed, EmBase, and Cochrane Library were systematically searched to identify eligible studies published throughout September 2021. The effect estimates with 95% confidence intervals (CIs) for gastric cancer morbidity or mortality in each study were applied to calculate the pooled results; these analyses were performed using the random-effects model. Twenty-six prospective cohort studies involving 4,956,350 individuals were selected; these studies reported 19,301 cases of gastric cancer and 2,871 cases of gastric cancer-associated mortality. High (RR: 1.25; 95%CI: 1.10–1.41; *P* = 0.001) or moderate (RR: 1.20; 95%CI: 1.04–1.38; *P* = 0.012) salt intake was associated with a greater risk of gastric cancer. High pickled food intake was associated with an increased gastric cancer risk (RR: 1.28; 95%CI: 1.05–1.57; *P* = 0.017), while moderate pickled foods intake had no significant effect on gastric cancer risk (RR: 1.10; 95%CI: 0.88–1.37; *P* = 0.390). Neither high (RR: 1.14; 95%CI: 0.95–1.36; *P* = 0.161) nor moderate (RR: 1.10; 95%CI: 0.87–1.40; *P* = 0.436) salted fish intake were associated with gastric cancer risk. A high intake of processed meat was significantly associated with a higher risk of gastric cancer (RR: 1.24; 95%CI: 1.03–1.49; *P* = 0.023), while moderate processed meat intake had no significant effect on the gastric cancer risk (RR: 1.01; 95%CI: 0.92–1.11; *P* = 0.844). High (RR: 1.04; 95%CI: 0.90–1.19; *P* = 0.626) and moderate (RR: 1.02; 95%CI: 0.94–1.11; *P* = 0.594) miso-soup intake had no effects on the gastric cancer risk. High intakes of salt, pickled food, and processed meat are associated with significantly increased risks of gastric cancer; these increased risks are also seen when participants consumed moderate amounts of salt.

## Introduction

Gastric cancer is the fifth most common type of cancer and is the third leading cause of cancer-related deaths worldwide (1). There were more than one million new cases of gastric cancer diagnosed in 2018, and the number of gastric cancer-related deaths reached 783,000 (1). Nearly 70% of new gastric cancer cases occurred in developing countries, especially, in China. Therefore, additional potential risk factors for this condition should be identified for preventing its progression. Studies have already found several lifestyle-associated factors could prevent the risk of gastric cancer, including the intake of citrus fruits (2), flavonols (3), dietary nitrates, nitrites, nitrosamines (4), a Mediterranean diet (5), dairy products (6), vitamin A, vitamin C, vitamin E (7), cruciferous vegetables (8), and dietary fiber (9), and physical activity (10). Moreover, several potential risk factors for gastric cancer, including intake of coffee, dietary fat, red meat, obesity, and smoking, have been identified (11–15). However, other dietary factors should be identified to further prevent the risk of gastric cancer.

Previous study have already demonstrated that increased dietary sodium intake is a modifiable risk factor for health (16). They point out reduced sodium intake significantly reduced blood pressure without any significant effects on blood lipids, catecholamine levels, and renal function for non-acutely ill adults. Moreover, reduced sodium intake was associated with a reduced risk of stroke and fatal coronary heart disease in adults. The World Health Organization currently recommends a salt intake of <2 g/d, a level that is largely based on a relatively small and short-term clinical trials evaluating the effects of moderate salt intake in the general population (17). Several systematic review and meta-analyses have illustrated the association of salt intake with the risk of gastric cancer (18–21). Excessive salt intake plays a dual effect at the initial stages, including gastritis and atrophy. Moreover, it might play an important role on the later stages of carcinogenesis through intestinal metaplasia and dysplasia stages (22). However, whether the strength of this association differs according to various characteristics in individuals remains unclear. Clarifying the optimal salt intake in the general population for preventing gastric cancer is particularly important, as this has not yet been definitively determined. Therefore, in the present study, we performed a systematic review and meta-analysis of prospective cohort studies to assess the strength of the association of dietary salt intake with the risk of gastric cancer; further, the comparison of this association in individuals with various characteristics was performed.

## Methods

### Data Sources, Search Strategy, and Selection Criteria

The Meta-analysis of Observational Studies in Epidemiology guidelines were applied to perform and report this systematic review and meta-analysis (23). Studies designed as prospective cohort studies and those that assessed the association of dietary salt intake with the risk of gastric cancer were eligible for inclusion in our study, and the publication language was restricted to English. PubMed, EmBase, and Cochrane Library were searched for articles published throughout September 2021, using (“Salt” OR “Salty” OR “Salted” OR “Sodium” OR “Diet” OR “Dietary” OR “Food” OR “Snack” OR “Bread” OR “Miso” OR “Pickle” OR “Processed fish” OR “Processed meat” OR “Salty fish)” AND (“Stomach cancer” OR “Gastric cancer)” AND “prospective” AND “human” AND “English” as the search terms. The reference lists of relevant original articles were also manually reviewed to identify any new eligible studies.

The literature search and study selection were independently undertaken by two authors, and any disagreements between these two authors were settled by mutual discussion until a consensus was reached. The inclusion criteria were as follows: (1) Study design: the study had to have a prospective cohort design; (2) Exposure: total dietary salt intake, pickled food, salted fish, processed meat, and miso-soup; (3) Control: the lowest intake of salt or a specific food category; (4) Outcome: gastric cancer-associated morbidity or mortality; and (5) Participants: general population or individuals without gastric cancer at inclusion. Retrospective cohort, traditional case-control, and series studies were excluded because the results of these studies may be susceptible to biases resulting from various confounding factors.

### Data Collection and Quality Assessment

Two authors (DY and SY) independently performed the data extraction and quality assessment, and any conflicts between these authors were examined and adjudicated by an additional author (GZ) by referring to the original studies. The collected characteristics and data included the study group or first author's name, publication year, country, sample size, age of participants, numbers of men and women, number of cases showing gastric cancer-associated morbidity or mortality, number of dietary questionnaire items, follow-up duration, reported effect estimates and 95% CI values, and covariates in the fully adjusted model. For studies that reported several multivariable adjusted effect estimates, the effect estimate that was maximally adjusted for potential confounders was used. The Newcastle-Ottawa Scale (NOS) was utilized to assess the methodological quality, which is quite comprehensive and has been partially validated for evaluating the quality of observational studies in meta-analyses (24). The NOS, based on selection [4 items (four stars): representativeness of the exposed cohort, selection of the non-exposed cohort, ascertainment of salt consumption, and demonstration that outcomes was not present at start of study], comparability [one item (two stars): comparability on the basis of the design or analysis], and outcome [three items (three stars): assessment of outcome, adequate follow-up duration, and adequate follow-up rate], and the “star system,” ranged from 0 to 9 for each study.

### Statistical Analysis

The association of the intake of salt or specific foods (pickled food, salted fish, processed meat, and miso-soup) with the risk of gastric cancer-associated morbidity or mortality was analyzed based on the effect estimate [risk ratio (RR), hazard ratio (HR), or odds ratio (OR)] and its 95% CI published in each study. The categories for salt or specific foods were divided based on tertiles, and the random-effects model was utilized to calculate the pooled RRs and 95% CIs for high or moderate vs. low salt or specific food intake (25). The *I*^2^ and Cochren Q statistic were used to assess heterogeneity across the included studies; significant heterogeneity was defined at *I*^2^ > 50.0% or *P* < 0.10 (26, 27). The stability of the pooled conclusions was assessed using sensitivity analyses through the sequential removal of each individual study (28). Stratified analyses were performed for high or moderate salt or specific foods on gastric cancer risk according to the country, gender, reported outcomes, follow-up duration, and adjustment for educational level, body mass index (BMI), alcohol, smoking, or physical activity (PA); further, the ratio between subgroups were compared based on the RRs and 95%CIs in each subset (29). Publication biases were assessed using both qualitative and quantitative methods, including funnel plots, and the Egger and Begg tests (30, 31). The inspection levels were two-sided for pooled results, and differences with *P* < 0.05 were regarded statistically significant. The STATA software was used to perform all the statistical analyses in this study (version 10.0; Stata Corporation, College Station, TX, USA).

## Results

### Literature Search

**Figure 1:**
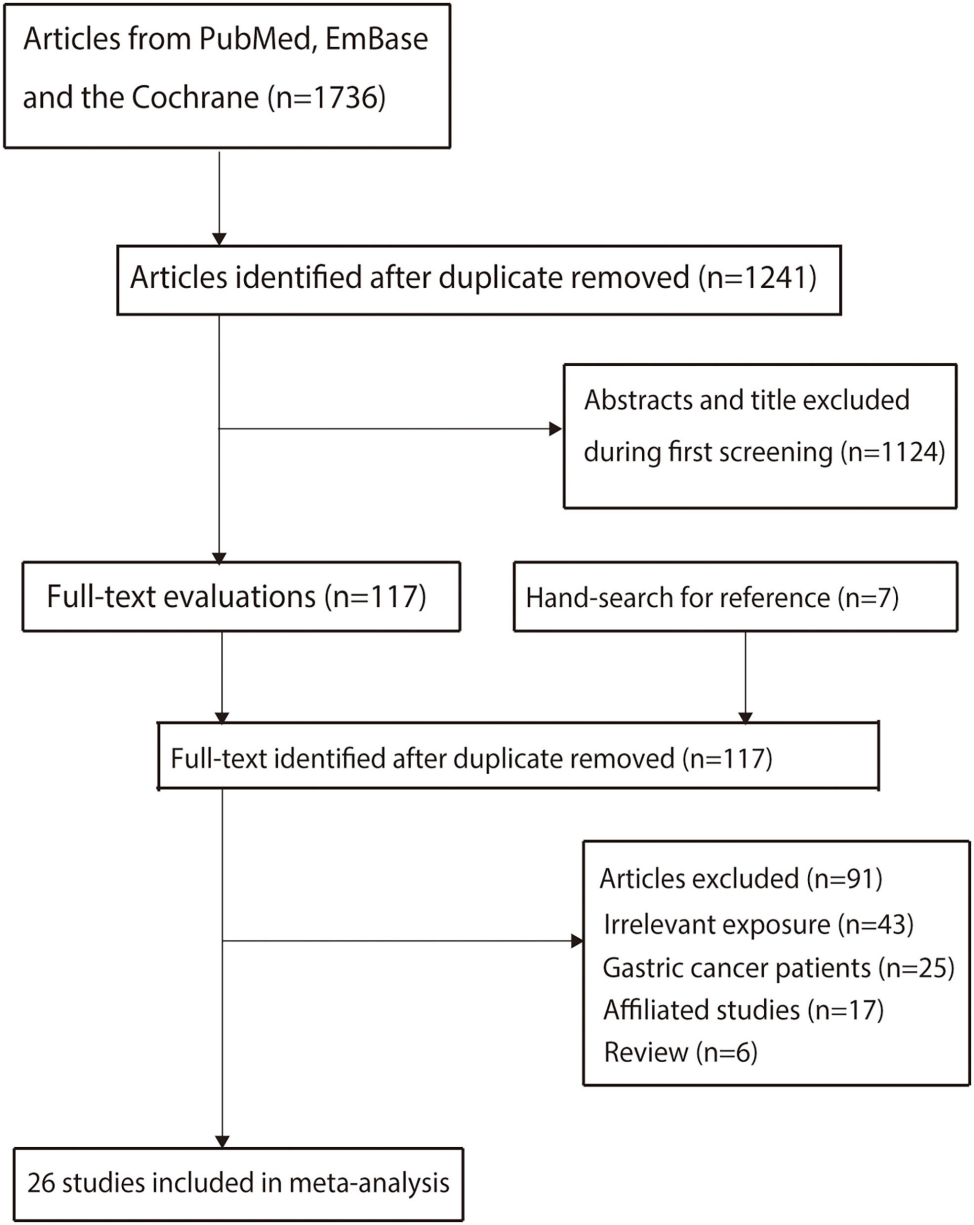
PRISMA flowchart of the literature search and study selection process.

A total of 1,736 articles were identified in electronic searches, and 1,241 studies were retained after duplicate articles were removed. Further, 1,124 studies were excluded because these studies contained irrelevant titles and abstracts. The remaining 117 studies were examined for further full-text evaluations, and 91 studies were excluded because: they contained irrelevant exposure (*n* = 43), they contained pre-existing gastric cancer patients (*n* = 25), they were affiliate studies (*n* = 17), and they were reviews (*n* = 6). A review of the reference lists of the relevant studies did not find any new eligible study. Finally, 26 prospective cohort studies were selected for the final meta-analysis (32–57); the flowchart representing the study selection process is shown in Figure 1.

### Study Characteristics

The baseline characteristics of the studies and participants are summarized in Table 1. A total of 4,956,350 individuals were recruited from 26 studies, and 19,301 cases of gastric cancer and 2,871 cases of gastric cancer-associated mortality were reported. The follow-up duration for each study ranged from 4.4 to 24.0 years, and 260–2,248,129 individuals were included in each study. Sixteen studies were performed in Asia, six studies were performed in Europe, and the remaining four studies were performed in the US. Eighteen studies reported the association of the intake of salt or specific foods with the risk of gastric cancer incidence, and nine studies reported the association of the intake of salt or specific foods with the risk of gastric cancer-associated mortality. Eleven studies showed an NOS score of eight stars, 10 studies showed an NOS score of seven stars, and the remaining five studies showed an NOS score of six stars.

**Table 1:** Baseline characteristic of studies included in meta-analysis.

**Study**	**Country**	**Sample size**	**Age (year)**	**Gender (men/women)**	**No of GC cases**	**Salt questionnaire**	**Follow-up (year)**	**Adjusted factors**	**NOS score**
JHCS 1990 (32)	Japan	7,990	> 45.0	7,990/0	Incidence (150)	17 items	17.5	Age	7
Kneller 1991 (33)	Norway	17,633	>35.0	17,633/0	Mortality (75)	35 items	20.0	Years of birth and smoking	7
Kato 1992 (34)	Japan	3,914	> 45.0	1,851/2,063	Incidence (45)	10 items	4.4	Age, sex, and residence	6
Kato 1992 (35)	Japan	9,753	> 30.0	NA	Mortality (57)	25 items	5.7	Age, and sex	6
HHSP 1998 (36)	US	11,907	46.4	5,610/6,297	Incidence (108)	13 items	14.8	Age, education, Japanese place of birth (for men added smoking and alcohol)	8
Knekt 1999 (37)	Finland	9,985	>15.0	5,274/4,711	Incidence (68)	NA	24.0	Sex, age, municipality, smoking and TE	7
CPS II 2001 (38)	US	970,045	56.0	436,654/533,391	Mortality (1,349)	32 items	14.0	Age, education, smoking, BMI, multivitamin and vitamin C use, aspirin use, race, and family history	8
Ngoan 2002 (39)	Japan	13,250	52.7	5,917/7,333	Mortality (116)	254 items	8.8	Age, gender, smoking, processed meat, liver, cooking or salad oil, suimono and pickled fruit	7
TNCS 2003 (40)	Netherlands	120,852	55.0–69.0	58,279/62,573	Incidence (282)	150 items	6.3	Age, gender, smoking, education, family history of stomach disorders and GC	8
Khan 2004 (41)	Japan	3,158	>40.0	1,524/1,634	Mortality (51)	37 items	14.3	Age, and smoking	6
CGCS group 2004 (42)	China	1,630	42.2	880/750	Incidence (18)	NA	7.5	Active treatment	8
JACC 2005 (43)	Japan	110,792	40.0–79.0	NA	Mortality (859)	33 items	12.0	Age	7
LSS 2005 (44)	Japan	38,576	34.0–98.0	14,885/23,691	Incidence (1,280)	22-items	20.0	Sex, sex-specific age, city, radiation dose, smoking, and education level	7
LGPT 2005 (45)	China	29,584	40.0–69.0	13,313/16,271	Incidence (1,452)	9 items	15.0	Age, gender, or smoking	8
Kurosawa 2006 (46)	Japan	8,035	> 30.0	3,652/4,383	Mortality (76)	29 items	11.0	Age, gender	7
THS 2006 (47)	Japan	2,467	57.9	1,023/1,444	Incidence (93)	70 items	14.0	Age, gender, *H pylori* infection, atrophic gastritis, history of peptic ulcer, family history of cancer, BMI, DM, TC, PA, alcohol, smoking and dietary factors (TE, TP, carbohydrate, B1-B2-C vitamin and dietary fiber)	7
SMC 2006 (48)	Sweden	61,433	53.4	0/61,433	Incidence (156)	67 items	18.0	Age, education, BMI, TE, alcohol, fruits, and vegetables	8
EPIC 2006 (49)	Europe	521,457	51.7	153,447/368,010	Incidence (330)	88–266 items	6.5	Sex, height, weight, education level, smoking, work and leisure PA, alcohol, TE, vegetable, citrus fruit, non-citrus fruit intake, red meat, and poultry	8
Sjodahl 2008 (50)	Norway	73,133	49.0	35,955/37,178	Incidence (313)	NA	15.4	Age, smoking, alcohol, PA and occupation	6
Kim 2010 (51)	Korea	2,248,129	30.0–80.0	1,420,981/827,148	Incidence (12,393)	13 items	7.0	Age, sex, BMI, smoking, alcohol, PA, and family history of cancer	7
JPHC 2010 (52)	Japan	77,500	45.0–74.0	35,730/41,770	Incidence (867)	138 items	7.7	Sex, age, BMI, smoking, alcohol, PA, and quintiles of energy, potassium, and calcium	8
Murata 2010 (53)	Japan	6,830	50.8	3,074/3,756	Mortality (87)	NA	13.9	Age, BMI, PA, smoking, alcohol, DM, vegetable, fruit, tea, red meat and processed meat	8
NIH-AARP 2011 (54)	US	337,074	50.0–71.0	177,792/159,282	Incidence (955)	124 item	10.0	Age, sex, BMI, education, ethnicity, smoking, alcohol, PA, and the daily intake of fruit, vegetables, saturated fat	8
SCHS 2017 (55)	Singapore	63,257	45.0–74.0	29,220/34,037	Incidence (691)	165 items	16.9	Age, interview year, father's dialect, gender, and education	8
Thapa 2019 (56)	US	260	43.8	88/172	Incidence (10)	NA	11.0–12.0	Age, sex, car ownership, and fruit and vegetable intake	6
KoGES and KMCC 2020 (57)	Korea	196,384/11,322	> 40.0 />20.0	NA	Mortality (201)/ Incidence (90)	103 items	7.4/13.3	Age, sex, survey year, BMI, smoking, and alcohol	7

*GC, gastric cancer; BMI, body mass index; DM, diabetes mellitus; TC, total cholesterol; PA, physical activity; TE, total energy; TP, total protein*.

### Salt Intake and Gastric Cancer Risk

**Figure 2:**
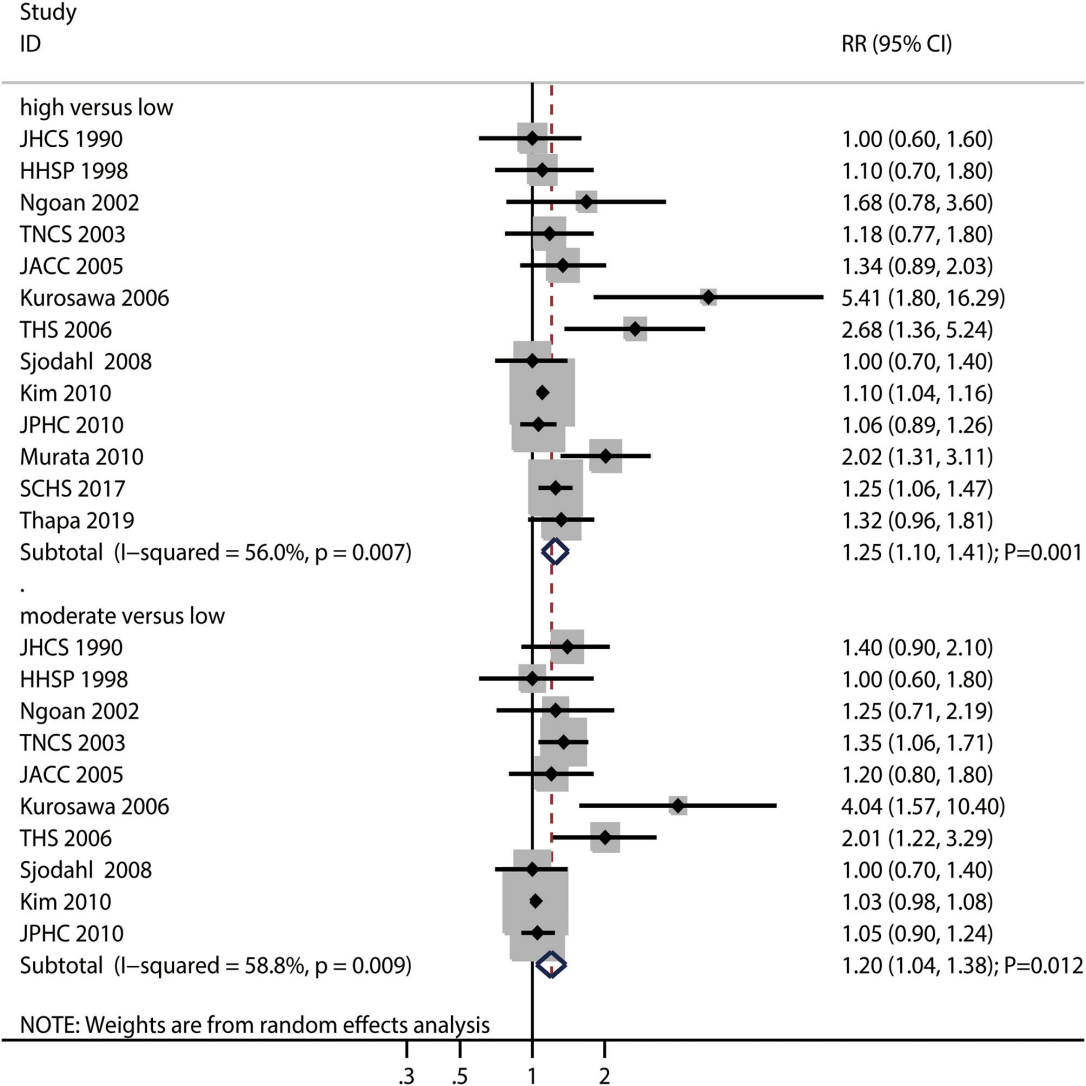
Association between high or moderate salt intake and subsequent gastric cancer risk.

The numbers of studies reporting the risk of gastric cancer with regard to high and moderate salt intake were 13 and 10, respectively. We noted that high (RR: 1.25; 95%CI: 1.10–1.41; *P* = 0.001) or moderate (RR: 1.20; 95%CI: 1.04–1.38; *P* = 0.012) salt intake were associated with a greater risk of gastric cancer (Figure 2).

There was significant heterogeneity for high (*I*^2^ = 56.0%; *P* = 0.007) and moderate (*I*^2^ = 58.8%; *P* = 0.009) salt intake among the included studies. Sensitivity analyses indicating the pooled conclusions for gastric cancer risk with regard to high and moderate salt intake are robust and not affected by any specific study (Supplemental 1). Subgroup analysis found that the gastric cancer risk related to high salt intake increased significantly in most subgroups, while high salt intake was not associated with the risk of gastric cancer if the pooled studies were performed in US or Europe and included female individuals. In case of the gastric cancer risk related to high salt intake, the gastric cancer incidence was lower than the gastric cancer mortality; the association between gastric cancer risk and high salt intake after ≥ 10.0 years of follow-up was greater than that observed after <10.0 years of follow-up (Table 2). In addition, the subgroup analysis indicated that moderate salt intake was associated with an increased risk of gastric cancer in case of pooled trials performed in Asia, studies reporting gastric cancer incidence, and studies involving a follow-up duration of ≥ 10.0 years, irrespective of the educational level status and adjustment for BMI, alcohol intake, and PA. Moreover, the strength of the association of gastric cancer risk and moderate salt intake was lower in studies with adjustment for alcohol intake than in studies without adjustment for alcohol intake (Table 3).

**Table 2:** Subgroup analysis for high vs. low salt intake and the risk of gastric cancer.

**Group**	**RR and 95%CI**	***P*-value**	**Heterogeneity (%)**	***P*-value for heterogeneity**	**Ratio between subgroups**	***P*-value for interaction test**
Country						
US or Europe	1.16 (0.96–1.40)	0.130	0.0	0.706	0.88 (0.68–1.13)	0.315
Asia	1.32 (1.11–1.55)	0.001	69.0	0.001		
Gender						
Men	1.10 (1.03–1.17)	0.002	0.0	0.812	1.01 (0.88–1.16)	0.898
Women	1.09 (0.96–1.23)	0.171	0.0	0.750		
Outcomes						
GC incidence	1.14 (1.05–1.25)	0.003	25.0	0.222	0.60 (0.39–0.93)	0.022
GC mortality	1.89 (1.24–2.89)	0.003	50.6	0.108		
Follow-up duration (years)						
≥ 10.0	1.38 (1.12–1.69)	0.002	57.6	0.015	1.25 (1.01–1.55)	0.037
<10.0	1.10 (1.04–1.16)	<0.001	0.0	0.692		
Adjusted educational						
Yes	1.23 (1.06–1.42)	0.006	0.0	0.866	0.95 (0.76–1.18)	0.626
No	1.30 (1.10–1.54)	0.003	65.0	0.002		
Adjusted BMI						
Yes	1.32 (1.03–1.69)	0.026	79.0	0.003	1.07 (0.81–1.43)	0.630
No	1.23 (1.07–1.43)	0.004	22.2	0.246		
Adjusted alcohol						
Yes	1.21 (1.01–1.46)	0.036	65.9	0.012	0.93 (0.72–1.20)	0.581
No	1.30 (1.09–1.55)	0.003	28.1	0.214		
Adjusted smoking						
Yes	1.22 (1.04–1.42)	0.015	55.9	0.026	0.92 (0.70–1.22)	0.583
No	1.32 (1.04–1.66)	0.020	48.2	0.103		
Adjusted PA						
Yes	1.24 (1.01–1.52)	0.038	72.7	0.005	0.98 (0.75–1.26)	0.856
No	1.27 (1.09–1.49)	0.002	19.6	0.275		

*RR, relative risk; CI, confidence interval; GC, gastric cancer; BMI, body mass index; PA, physical activity*.

**Table 3:** Subgroup analysis for moderate vs. low salt intake and the risk of gastric cancer.

**Group**	**RR and 95%CI**	***P*-value**	**Heterogeneity (%)**	***P*-value for heterogeneity**	**Ratio between subgroups**	***P*-value for interaction test**
Country						
US or Europe	1.18 (0.95–1.46)	0.132	17.3	0.299	0.95 (0.72–1.26)	0.731
Asia	1.24 (1.03–1.49)	0.022	65.8	0.007		
Gender						
Men	1.04 (0.98–1.10)	0.217	0.0	0.812	1.04 (0.94–1.15)	0.437
Women	1.00 (0.92–1.08)	0.981	0.0	0.521		
Outcomes						
GC incidence	1.15 (1.00–1.31)	0.045	54.9	0.038	0.72 (0.40–1.31)	0.285
GC mortality	1.59 (0.89–2.83)	0.115	63.9	0.063		
Follow-up duration (years)						
≥ 10.0	1.38 (1.02–1.87)	0.036	57.8	0.037	1.27 (0.92–1.75)	0.152
<10.0	1.09 (0.97–1.21)	0.134	41.5	0.162		
Adjusted educational						
Yes	1.29 (1.03–1.60)	0.024	0.0	0.326	1.08 (0.82–1.43)	0.564
No	1.19 (1.01–1.40)	0.032	60.2	0.014		
Adjusted BMI						
Yes	1.12 (0.93–1.34)	0.242	71.2	0.031	0.88 (0.67–1.15)	0.358
No	1.27 (1.04–1.54)	0.018	32.1	0.183		
Adjusted alcohol						
Yes	1.07 (0.94–1.22)	0.283	42.8	0.136	0.77 (0.59–1.00)	0.048
No	1.39 (1.11–1.74)	0.004	28.1	0.234		
Adjusted smoking						
Yes	1.13 (0.99–1.28)	0.066	49.6	0.064	0.70 (0.42–1.18)	0.183
No	1.61 (0.97–2.66)	0.066	62.7	0.068		
Adjusted PA						
Yes	1.08 (0.94–1.26)	0.276	57.0	0.073	0.81 (0.62–1.04)	0.101
No	1.34 (1.08–1.65)	0.007	25.4	0.243		

*RR, relative risk; CI, confidence interval; GC, gastric cancer; BMI, body mass index; PA, physical activity*.

### Pickled Food Intake and Gastric Cancer Risk

**Figure 3:**
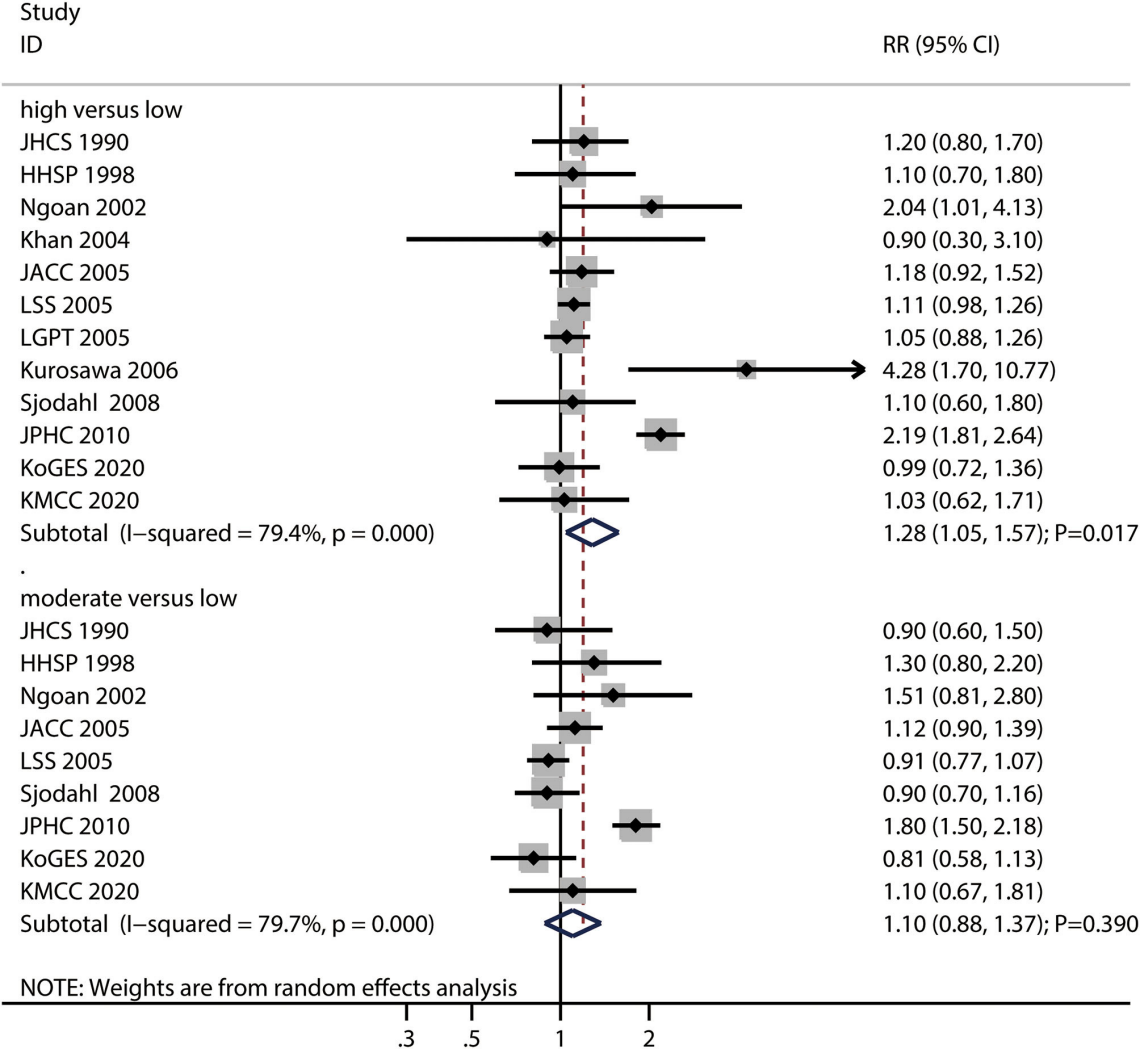
Association between high or moderate pickled food intake and subsequent gastric cancer risk.

The numbers of studies reporting the risk of gastric cancer related to high and moderate pickled food intake were 12 and nine, respectively. We noted that high pickled food intake was associated with an increased risk of gastric cancer (RR: 1.28; 95%CI: 1.05–1.57; *P* = 0.017), while moderate pickled food intake was not (RR: 1.10; 95%CI: 0.88–1.37; *P* = 0.390) (Figure 3).

Moreover, there was significant heterogeneity for gastric cancer risk related to high (*I*^2^ = 79.4%; *P* < 0.001) and moderate (*I*^2^ = 79.7%; *P* < 0.001) pickled food intake across the included studies. Sensitivity analyses found that the pooled conclusions for gastric cancer related to high and moderate pickled food intake were stable after the sequential exclusion of individual studies (Supplemental 1). The subgroup analysis demonstrated that high pickled food intake was associated with an increased risk of gastric cancer in case of pooled studies performed in Asia, studies with a follow-up of ≥ 10.0 years, and studies without adjustment for the educational level, BMI, alcohol intake, and PA (Supplemental 2). Moreover, the results of the subgroup analyses for moderate pickled food intake and gastric cancer risk were consistent with those of the overall analysis and remained statistically non-significant (Supplemental 2).

### Salted Fish Intake and Gastric Cancer Risk

**Figure 4:**
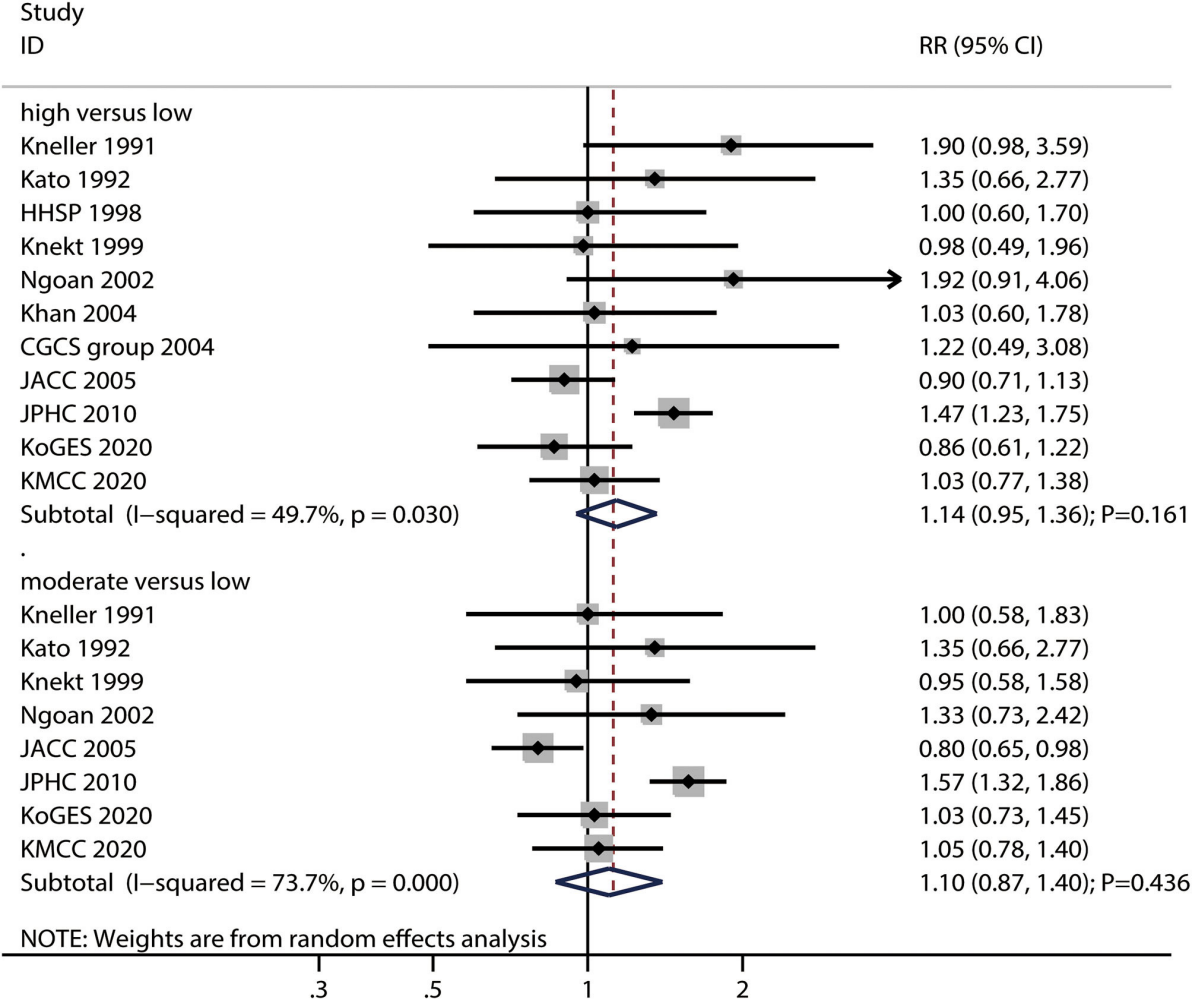
Association between high or moderate salted fish intake and subsequent gastric cancer risk.

The numbers of studies reporting the risk of gastric cancer related to high and moderate salted fish intake were 11 and eight, respectively. We noted that high (RR: 1.14; 95%CI: 0.95–1.36; *P* = 0.161) or moderate (RR: 1.10; 95%CI: 0.87–1.40; *P* = 0.436) salted fish intakes were not associated with the risk of gastric cancer (Figure 4), and a potential significant heterogeneity for gastric cancer risk related to high (*I*^2^ = 49.7%; *P* = 0.030) and moderate (*I*^2^ = 73.7%; *P* < 0.001) salted fish intake was noted among the included studies.

The pooled conclusions for gastric cancer risks related to high and moderate salted fish intake were robust and not affected by the exclusion of any particular study (Supplemental 1). The subgroup analysis found that high salted fish intake was associated with an increased risk of gastric cancer in case of pooled studies reporting gastric cancer incidence and studies with adjustment for the PA; moreover, the strength of the association of gastric cancer risk with high salted fish intake in studies with the adjustment for PA was higher than that in case of studies without the adjustment for adjusted PA (Supplemental 2). Moreover, the subgroup analysis found that moderate salted fish intake was associated with an increased risk of gastric cancer if the follow-up duration was <10.0 years, and in case for studies with the adjustment for PA, while moderate salted fish intake was associated with a reduced risk of gastric cancer in women. The differences between the subgroups in the analyses based on gender, follow-up duration, and adjustment for PA were statistically significant (Supplemental 2).

### Processed Meat Intake and Gastric Cancer Risk

**Figure 5:**
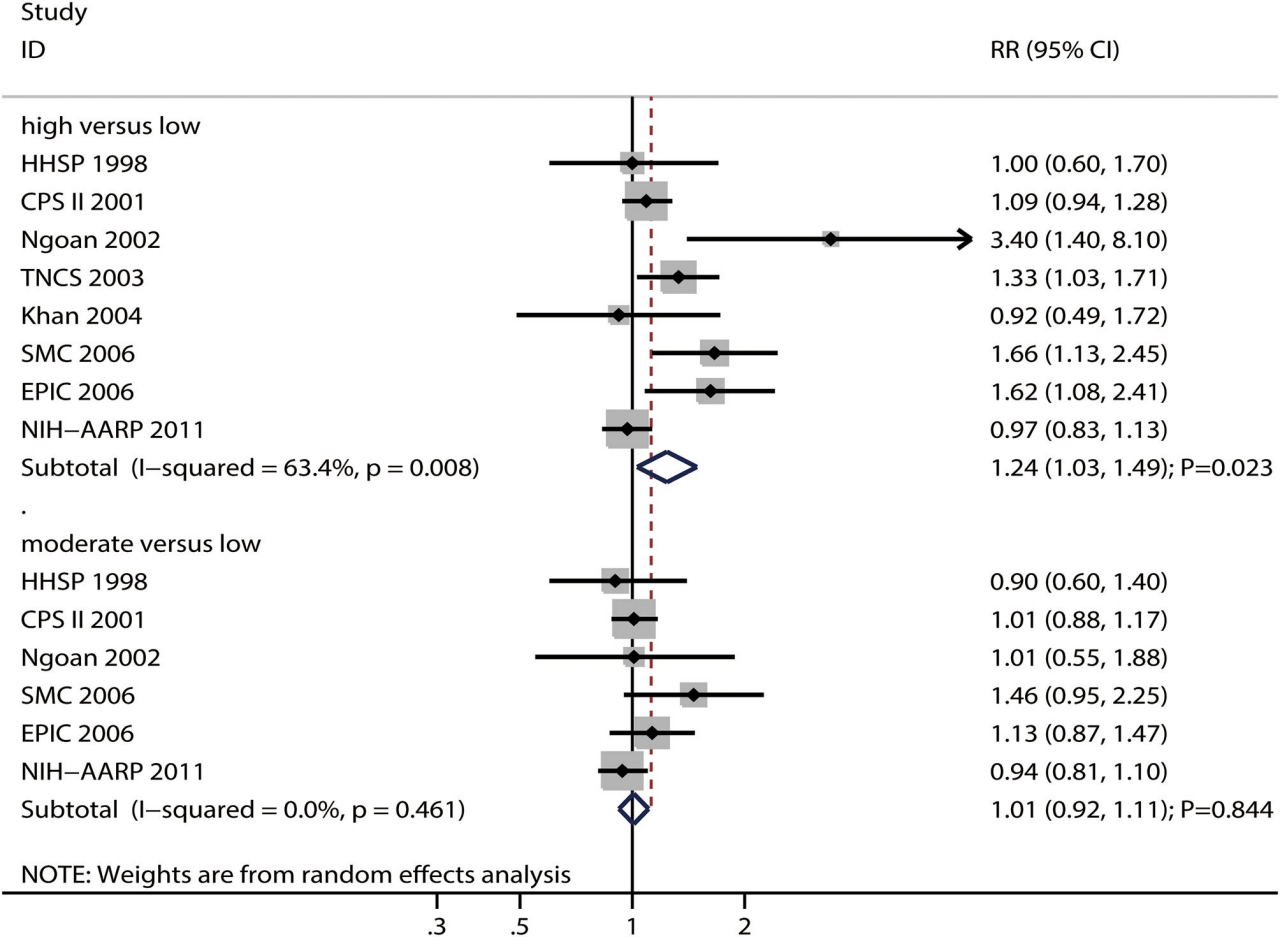
Association between high or moderate processed meat intake and subsequent gastric cancer risk.

The numbers of studies reporting the risk of gastric cancer related to high and moderate processed meat intake were eight and six, respectively. We noted that high processed meat intake was associated with an increased risk of gastric cancer (RR: 1.24; 95%CI: 1.03–1.49; *P* = 0.023), while moderate processed meat intake had no significant effect on the risk of gastric cancer (RR: 1.01; 95%CI: 0.92–1.11; *P* = 0.844) (Figure 5).

There was significant heterogeneity for gastric cancer risk related to high processed meat intake (*I*^2^ = 63.4%; *P* = 0.008), while no evidence of heterogeneity for gastric cancer risk related to moderate processed meat intake (*I*^2^ = 0.0%; *P* = 0.461) was noted. The pooled conclusions for gastric cancer risk related to high processed meat intake were variable, while the gastric cancer risk related to moderate processed meat intake was stable (Supplemental 1). The subgroup analyses showed that high processed meat intake was associated with an increased risk of gastric cancer in case of pooled studies performed in the US or Europe, studies with a follow-up duration of <10.0 years, studies with adjustment for educational level, and studies without adjustment for smoking and PA (Supplemental 2). Moreover, moderate processed meat intake was not associated with the risk of gastric cancer in all subgroups (Supplemental 2).

### Miso-Soup Intake and Gastric Cancer Risk

**Figure 6:**
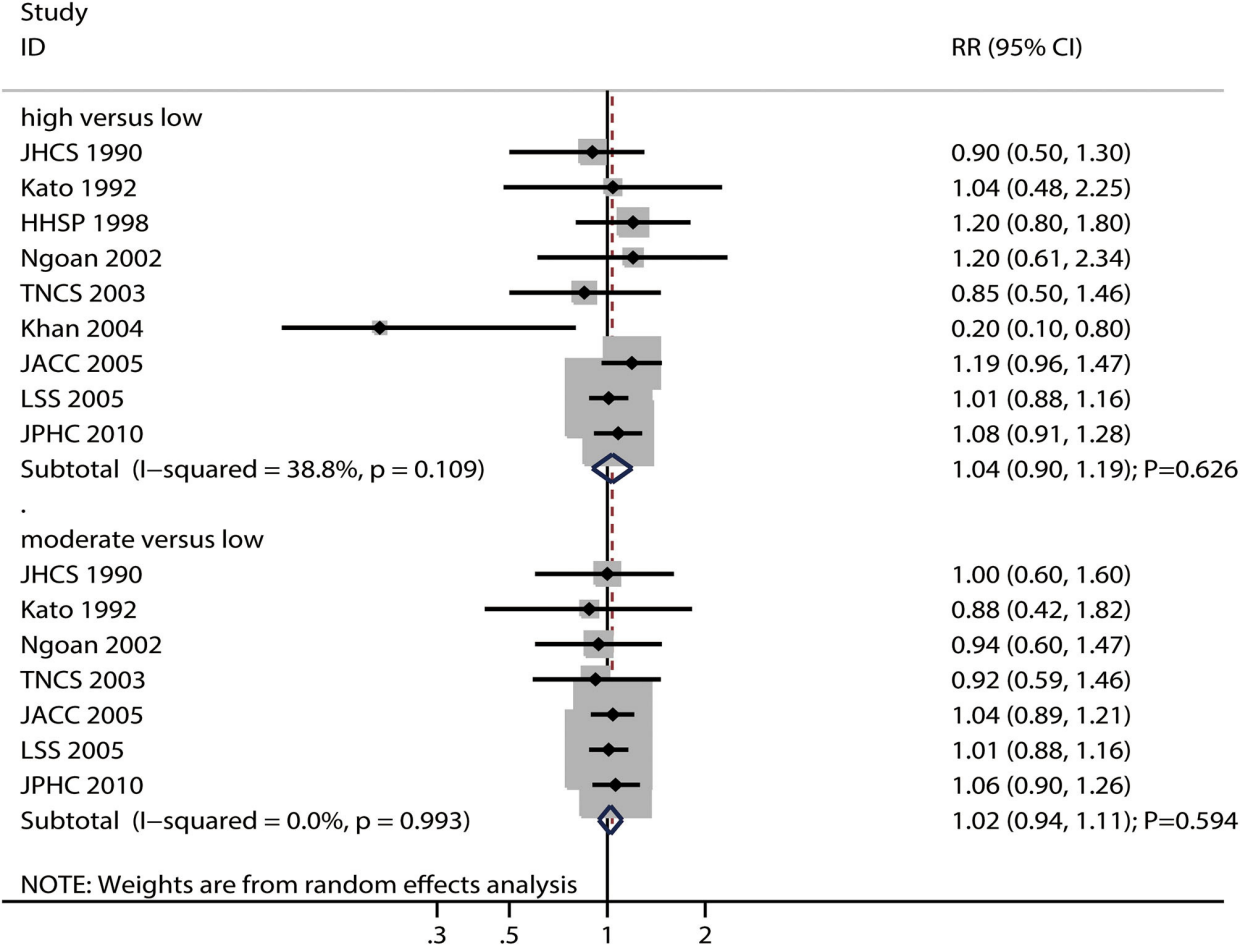
Association between high or moderate miso-soup intake and subsequent gastric cancer risk.

The numbers of studies reporting the risk of gastric cancer with regard to high and moderate miso-soup intake were nine and seven, respectively. We noted that high (RR: 1.04; 95%CI: 0.90–1.19; *P* = 0.626) and moderate (RR: 1.02; 95%CI: 0.94–1.11; *P* = 0.594) miso-soup intake were not associated with the risk of gastric cancer, and no significant heterogeneity for gastric cancer related to high (*I*^2^ = 38.8%; *P* = 0.109) and moderate (*I*^2^ = 0.0%; *P* = 0.993) miso-soup intake was observed (Figure 6). The pooled conclusions for gastric cancer risk related to high and moderate miso-soup intakes were found to be robust after the sequential removal of single studies (Supplemental 1). The results of the subgroup analyses showed that the gastric cancer risks related to high and moderate miso-soup intakes were consistent with the findings of the overall analysis in all subgroups (Supplemental 2).

### Publication Bias

Review of the funnel plots could not rule out the potential of publication bias for conclusions regarding high and moderate intake of salt or specific foods (Supplemental 3). We noted potential significant publication bias for gastric cancer risk related to high and moderate salt intake, but no significant publication biases for gastric cancer risk related to high and moderate pickled food, salted fish, processed meat, and miso-soup intakes. The conclusions remained unchanged after adjustment for publication bias for gastric cancer related to high and moderate salt intake using the trim and fill method (58).

## Discussion

Our study intended to assess the association of the intake of salt or specific foods with the risk of gastric cancer based on high-quality prospective cohort studies. A total of 4,956,350 individuals with 19,301 cases of gastric cancer and 2,871 cases of gastric cancer-associated mortality from 26 studies were identified and a broad range of characteristics of the studies or individuals were considered. The findings of this study found that high and moderate salt intakes increase the risk of gastric cancer. Moreover, high pickled food and processed meat intakes were associated with an increased risk of gastric cancer, while moderate pickled food and processed meat intakes were not. Furthermore, salted fish and miso-soup intakes were not associated with the risk of gastric cancer, irrespective of whether the intakes were high or moderate. The associations of salt or specific food intake with the risk of gastric cancer were affected by gender, reported outcomes, follow-up duration, and adjustment for alcohol intake and PA. Finally, considering the satisfactory quality of the included studies, the findings of this study are recommendable for the general population.

Several systematic review and meta-analyses have already addressed the potential role of dietary salt or specific foods in increasing the risk of gastric cancer (19, 21). A study conducted by D'Elia et al. found that high and moderate dietary salt intakes were associated with an increased risk of gastric cancer, and this association was stronger in case of Japanese populations and a higher consumption of selected salt-rich foods (19). Similarly, Ge et al. identified 11 studies and found that dietary salt intake was positively related to the risk of gastric cancer (21). However, stratified analyses performed on the basis of gender and adjustment for different parameters levels were not considered. Therefore, we performed this study to systematically assess the associations of salt or specific food intakes with the risk of gastric cancer.

Our study found that high or moderate salt intakes were associated with an increased risk of gastric cancer, which are consistent with the findings of previous meta-analyses (19, 21). Several potential mechanisms could explain the increase in the gastric cancer risk associated with the high intakes of salt, pickled food, and processed meat: (1) dietary salt was associated with N-methyl-N-nitro-N-nitrosoguanidine, which could induce carcinogenic effects in the stomach (59); (2) the mucosal barrier could be destroyed by high salt concentrations in the intragastric region, which may cause inflammation and damage, and subsequently, diffuse erosion and degeneration of the gastric mucosa. These symptoms could induce proliferous changes and enhance the effects of food-derived carcinogens (60); and (3) the mucosal damage could enhance *H. pylori* colonization in mice and humans, leading to chronic gastritis, which is associated with a greater risk of gastric cancer (61–63).

We noted that high pickled food and processed meat intakes increased the risk of gastric cancer, while moderate pickled food and processed meat intake did not affect the risk of gastric cancer. Further, increased intakes of salted fish and miso-soup did not affect the risk of gastric cancer. Several reasons could explain these results: (1) the follow-up duration for these studies were shorter than the duration needed to show a clinical benefit, resulting in broad confidence intervals and no statistically significant associations; (2) the items of food-frequency questionnaire across the included studies differed, which may introduce biases with regard to the association of the intake of salt or specific foods with the risk of gastric cancer; (3) the net effect estimates could be affected by the levels of salt or specific foods in the control arm; (4) the adjusted factors across the included studies are different, which may introduce biases with regard to the pooled results; and (5) the study quality and number of studies reported for each exposure are different, and thus, the robustness of pooled conclusions could be affected.

The subgroup analyses found that the potential associations of the intakes of salt or specific foods with the risk of gastric cancer could affected by gender, reported outcomes, follow-up duration, and adjustment for alcohol intake and PA. The potential reasons for these differences are: (1) gender, reported outcomes, and follow-up duration could affect gastric cancer incidence and gastric cancer-associated mortality, and the power to detect potential associations are different; and (2) alcohol intake and PA are significantly associated with the risk of gastric cancer; thus, complete adjustment for both these parameters should be performed to avoid potential confounding bias. Moreover, we noted that the associations of the intake of salt or specific foods with the risk of gastric cancer differed in various countries. The potential reason for this could be that Asia shows the highest incidence of gastric cancer in the world, i.e., over 4–7 times higher than that in the US or Europe; this could make it easier to detect the differences in this relationship in different regions (1).

Although our analysis is based on prospective cohort studies, several limitations of the present study should be acknowledged. First, the levels of adjustment for various parameters across the included studies differed; because these factors play an important role in the progression of gastric cancer, their adjustments must be consistent. Second, the differences in the food-frequency questionnaire could affect the level of exposure to each food type, which might introduce biases in the relationship between the intakes of dietary salt or specific foods and gastric cancer risk. Third, the dose-response analysis was restricted owing to the unavailability of cases and people or person–year data in each category. Fourth, there are inherent limitations associated with the analysis based on published articles, including inevitable publication bias and restricted detailed analyses.

## Conclusion

In summary, the results of the present study suggest that dietary salt intake may have harmful effects on the risk of gastric cancer in terms of gastric cancer-associated morbidity and mortality. Moreover, high pickled food and processed meat intakes were associated with an increased risk of gastric cancer. Further randomized controlled trials should be performed to assess the effects of reduced dietary salt intake on the risk of gastric cancer according to different characteristics of the subjects.

## Data Availability Statement

The original contributions presented in the study are included in the article/Supplementary Material, further inquiries can be directed to the corresponding author.

## Ethics Statement

Ethical review and approval was not required for the study on human participants in accordance with the local legislation and institutional requirements. Written informed consent for participation was not required for this study in accordance with the national legislation and the institutional requirements.

## Author Contributions

GZ: conception and design, administrative support, and provision of study materials or patients. DY: collection and assembly of data. SY and BW: data analysis and interpretation. GZ, DY, SY, and BW: manuscript writing and final approval of manuscript. All authors contributed to the article and approved the submitted version.

## Conflict of Interest

The authors declare that the research was conducted in the absence of any commercial or financial relationships that could be construed as a potential conflict of interest.

## Publisher's Note

All claims expressed in this article are solely those of the authors and do not necessarily represent those of their affiliated organizations, or those of the publisher, the editors and the reviewers. Any product that may be evaluated in this article, or claim that may be made by its manufacturer, is not guaranteed or endorsed by the publisher.
