# Supplementary material for: Dietary Salt Intake and Gastric Cancer Risk: A Systematic Review and Meta-Analysis
Source: Front Nutr. 2021 Dec 8;8:801228. doi: 10.3389/fnut.2021.801228 (PMC8692376; doi:10.3389/fnut.2021.801228)
Supplement: Supplementary file 1 [file Presentation_1.pdf]

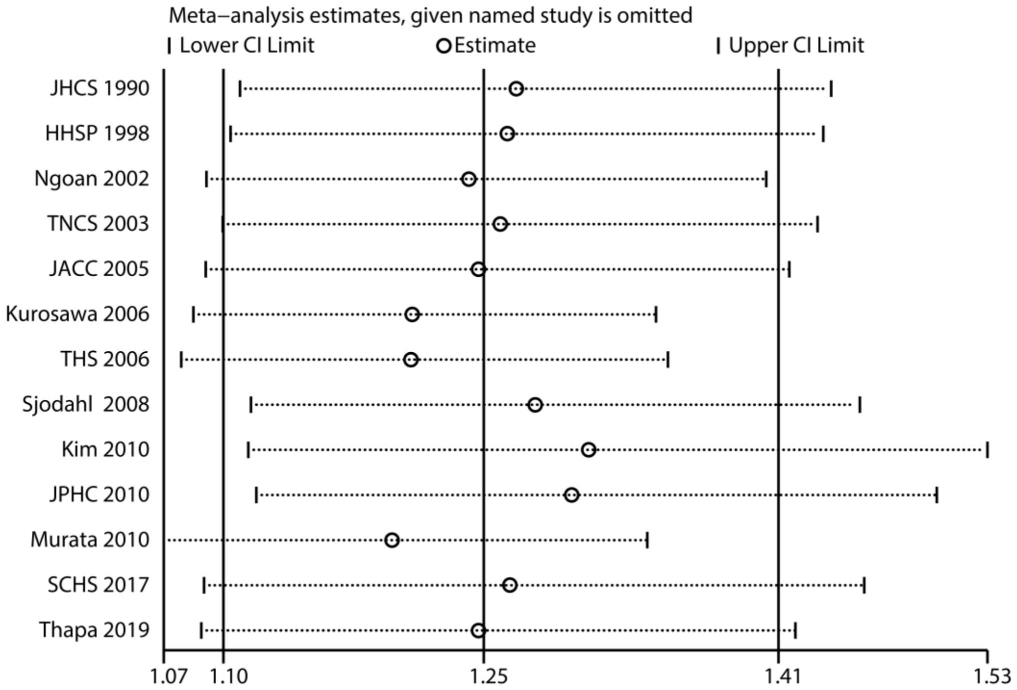

Figure S1. Sensitivity analysis for high versus low salt intake and the risk of gastric cancer

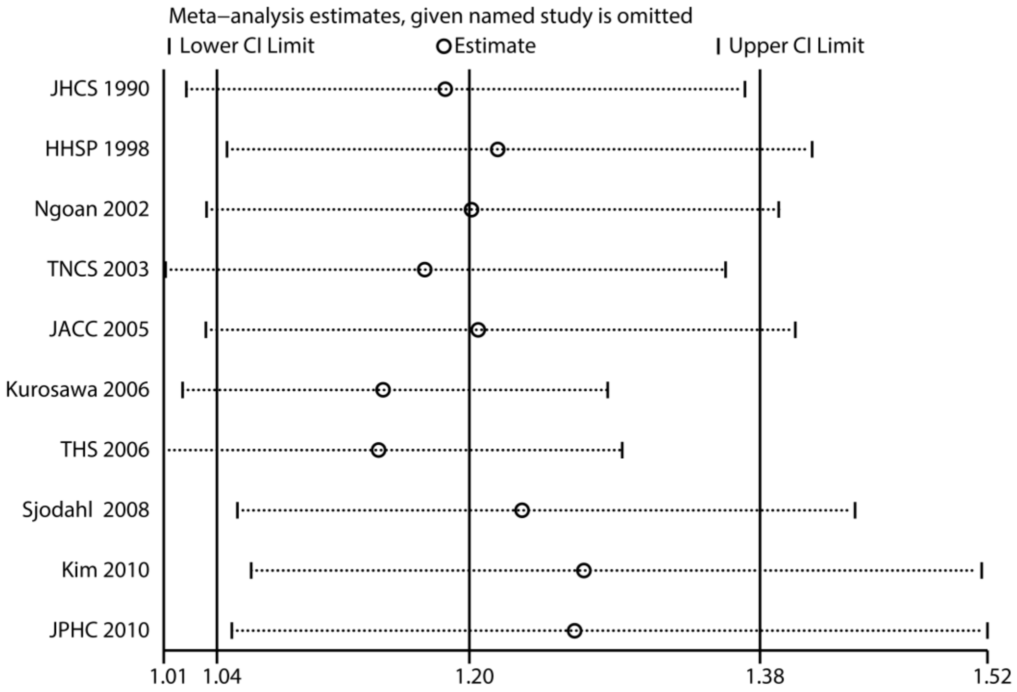

Figure S2. Sensitivity analysis for moderate versus low salt intake and the risk of gastric cancer

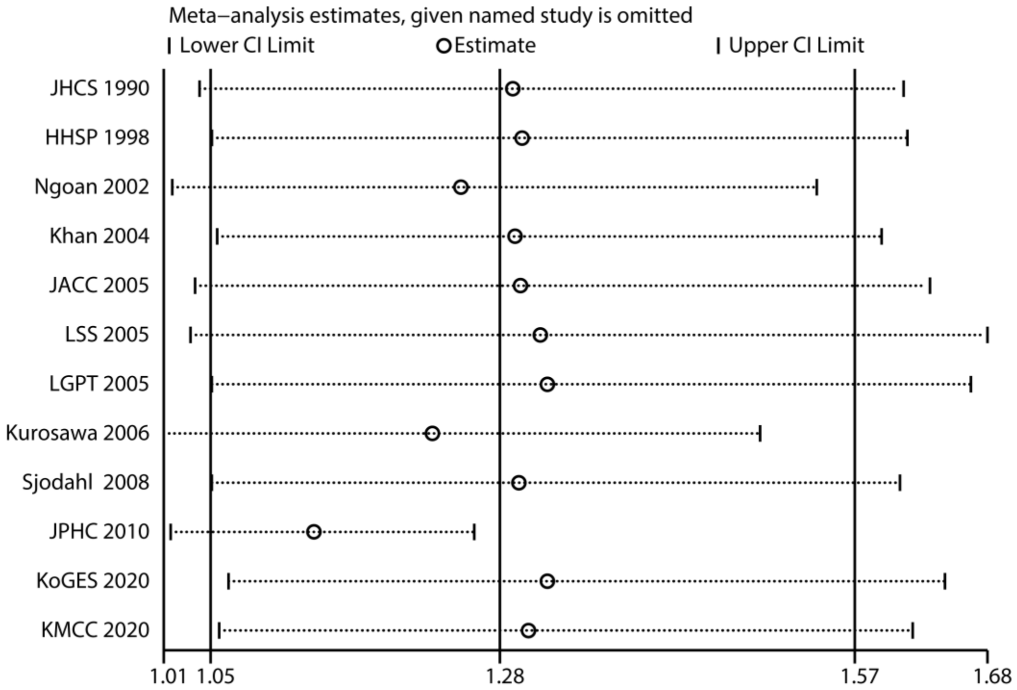

Figure S3. Sensitivity analysis for high versus low pickled food intake and the risk of gastric cancer

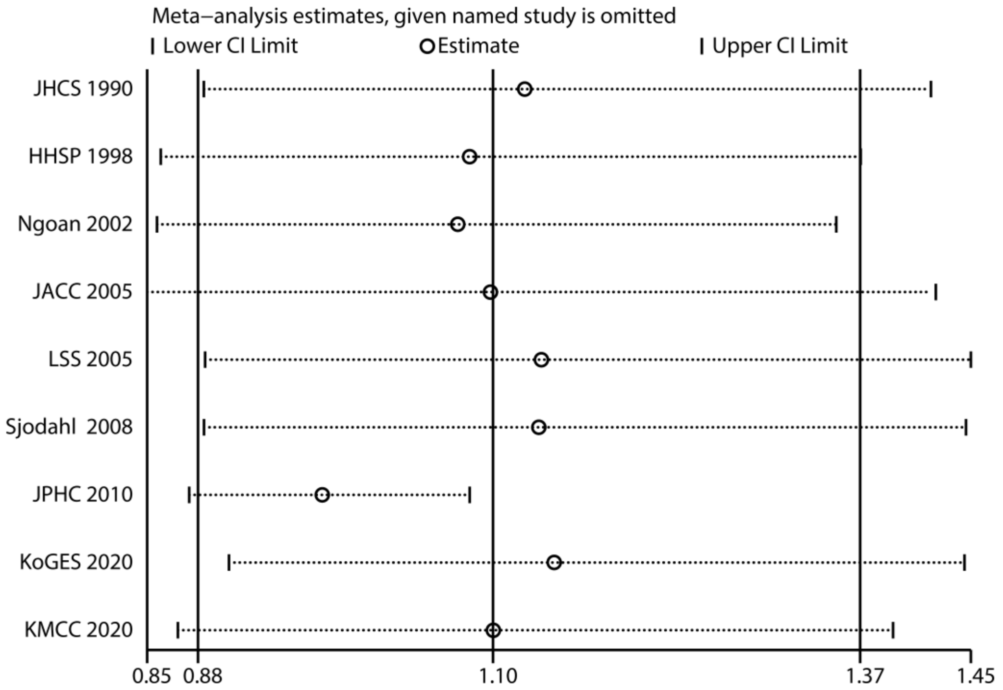

Figure S4. Sensitivity analysis for moderate versus low pickled food intake and the risk of gastric cancer

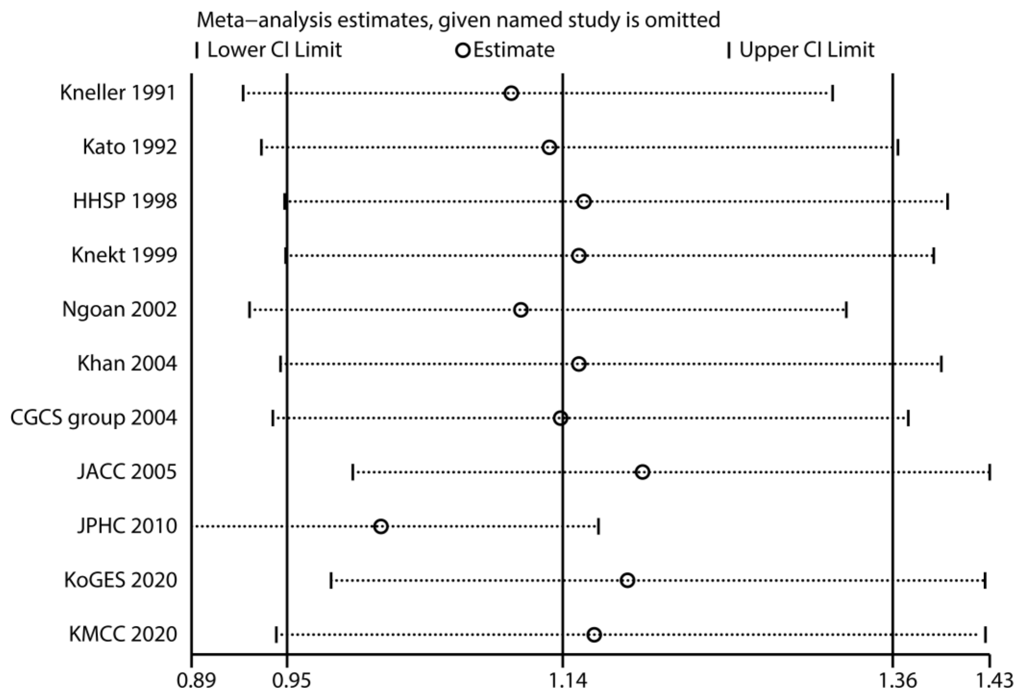

Figure S5. Sensitivity analysis for high versus low salted fish intake and the risk of gastric cancer

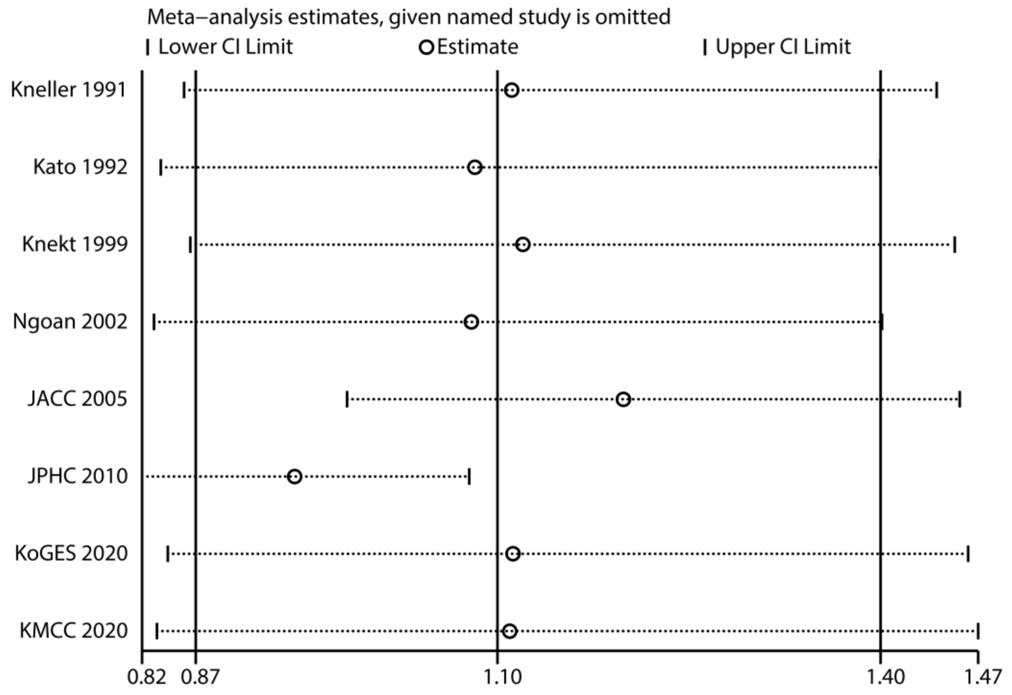

Figure S6. Sensitivity analysis for moderate versus low salted fish intake and the risk of gastric cancer

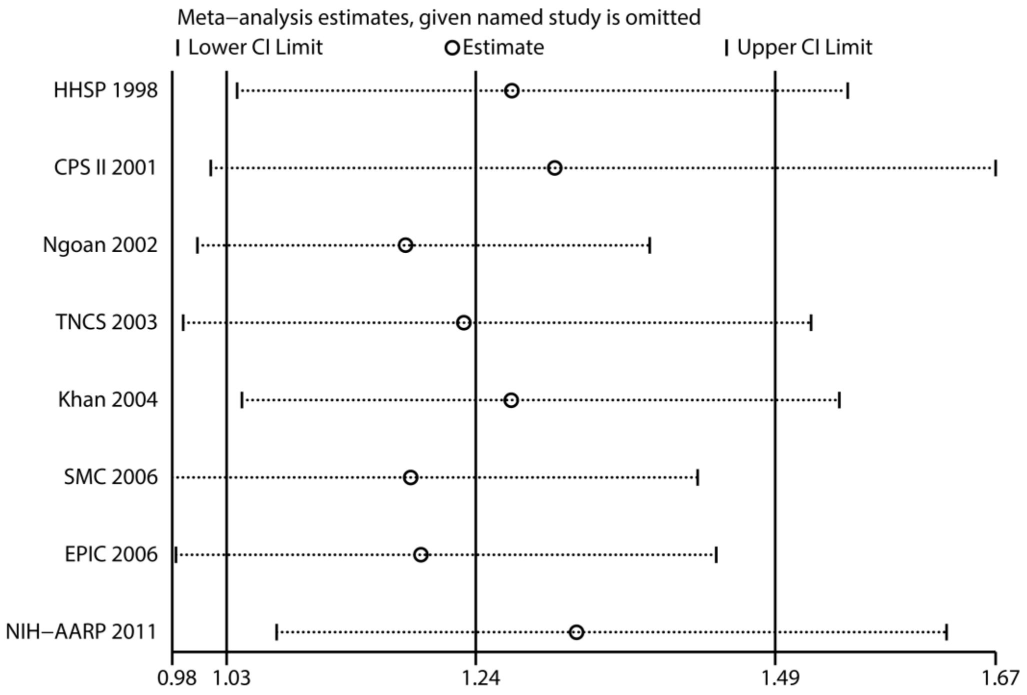

Figure S7. Sensitivity analysis for high versus low processed meat intake and the risk of gastric cancer

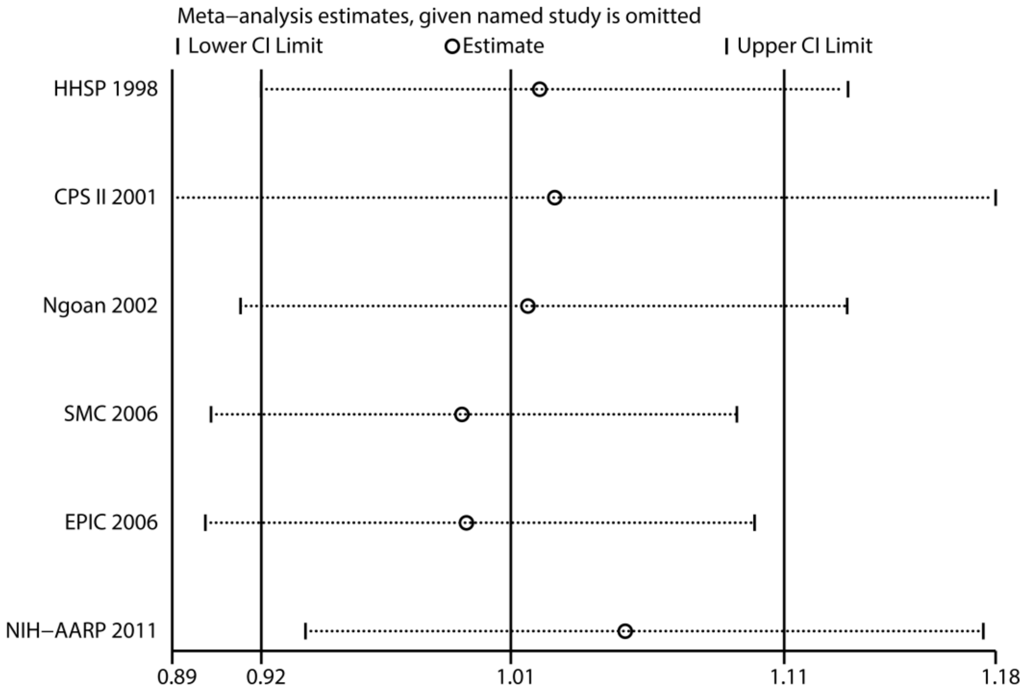

Figure S8. Sensitivity analysis for moderate versus low processed meat intake and the risk of gastric cancer

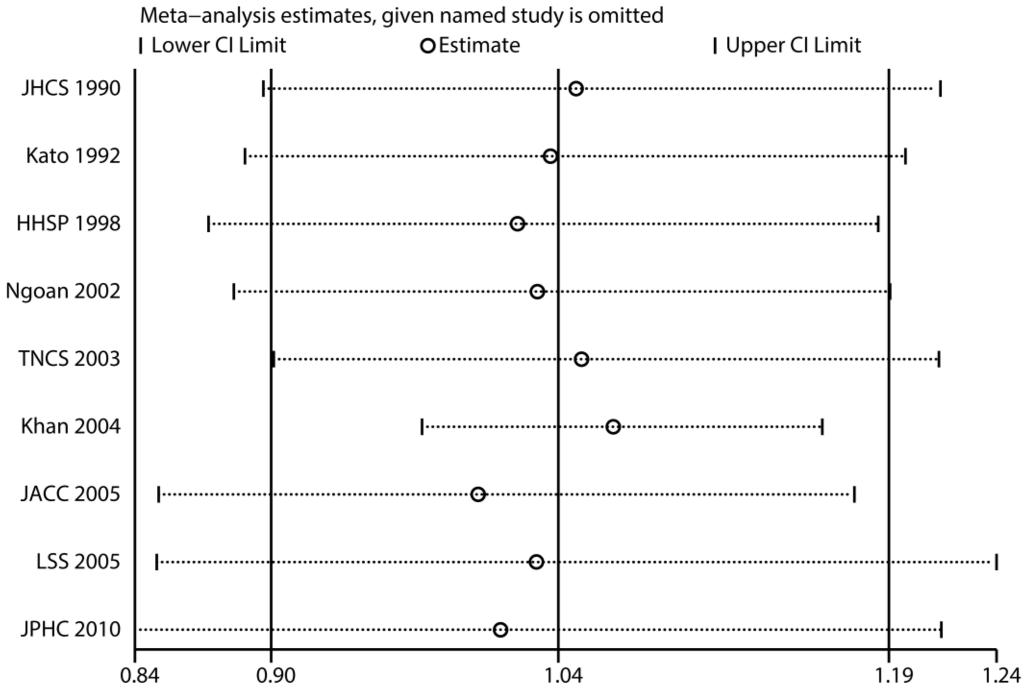

Figure S9. Sensitivity analysis for high versus low miso-soup intake and the risk of gastric cancer

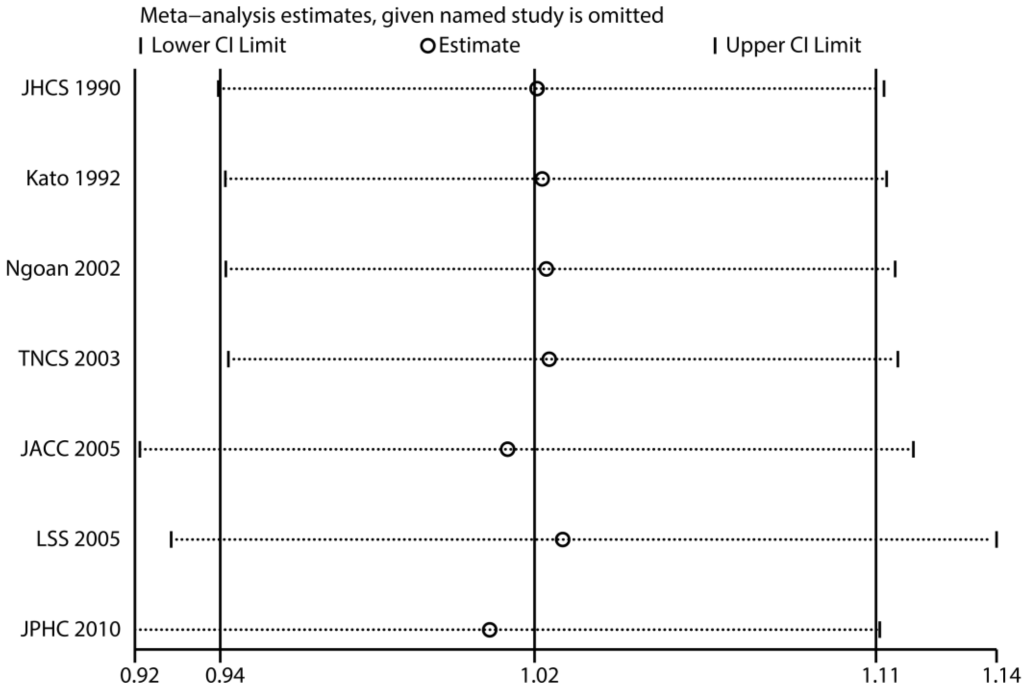

Figure S10. Sensitivity analysis for moderate versus low miso-soup intake and the risk of gastric cancer
