# Supplementary material for: Dietary Salt Intake and Gastric Cancer Risk: A Systematic Review and Meta-Analysis
Source: Front Nutr. 2021 Dec 8;8:801228. doi: 10.3389/fnut.2021.801228 (PMC8692376; doi:10.3389/fnut.2021.801228)
Supplement: Supplementary file 2 [file Presentation_2.pdf]

Table S1. Subgroup analysis for high versus low pickled food intake and the risk of gastric cancer

Group	RR and 95%CI	P value	Heterogeneity (%)	P value for heterogeneity	Ratio between subgroups	P value for interaction test
Country						
US or Europe	1.10 (0.77-1.57)	0.602	0.0	1.000	0.83	0.399
Asia	1.32 (1.05-1.66)	0.018	83.0	<0.001	(0.55-1.27)	
Gender						
Men	1.16 (0.92-1.48)	0.212	13.2	0.330	0.82	0.393
Women	1.42 (0.95-2.11)	0.089	0.0	0.922	(0.51-1.30)	
Outcomes						
GC incidence	1.24 (0.95-1.61)	0.112	85.7	< 0.001	0.89	0.613
GC mortality	1.40 (0.95-2.07)	0.089	64.0	0.025	(0.55-1.42)	
Follow-up duration (years)						
≥ 10.0	1.12 (1.01-1.25)	0.030	12.7	0.329	0.69	0.233
< 10.0	1.62 (0.89-2.94)	0.115	88.8	<0.001	(0.38-1.27)	
Adjusted educational						
Yes	1.11 (0.98-1.25)	0.094	0.0	0.971	0.83	0.206
No	1.34 (1.03-1.75)	0.030	81.0	< 0.001	(0.62-1.11)	
Adjusted BMI						
Yes	1.34 (0.73-2.45)	0.348	90.8	< 0.001	1.15	0.668
No	1.17 (1.02-1.33)	0.026	32.2	0.160	(0.62-2.13)	
Adjusted alcohol						
Yes	1.25 (0.82-1.91)	0.303	84.9	< 0.001	1.04	0.860
No	1.20 (1.01-1.41)	0.036	49.0	0.067	(0.66-1.64)	
Adjusted smoking						
Yes	1.24 (0.97-1.58)	0.092	82.7	< 0.001	0.84	0.516
No	1.48 (0.92-2.38)	0.102	71.7	0.029	(0.49-1.43)	
Adjusted PA						
Yes	1.63 (0.84-3.18)	0.150	81.5	0.020	1.43	0.300
No	1.14 (1.01-1.28)	0.038	28.1	0.186	(0.73-2.81)	

Table S2. Subgroup analysis for moderate versus low pickled food intake and the risk of gastric cancer

Group	RR and 95%CI	P value	Heterogeneity (%)	P value for heterogeneity	Ratio between subgroups	P value for interaction test
Country						
US or Europe	1.01 (0.72-1.41)	0.951	38.5	0.202	0.90 (0.59-1.38)	0.636
Asia	1.12 (0.86-1.46)	0.413	83.3	< 0.001		
Gender						
Men	1.06 (0.87-1.30)	0.543	0.0	0.496	0.75 (0.50-1.13)	0.167
Women	1.41 (0.99-2.00)	0.055	0.0	0.673		
Outcomes						
GC incidence	1.12 (0.82-1.52)	0.479	85.6	< 0.001	1.07 (0.70-1.62)	0.764
GC mortality	1.05 (0.79-1.40)	0.745	49.8	0.136		
Follow-up duration (years)						
≥ 10.0	0.98 (0.88-1.09)	0.700	0.0	0.520	0.75 (0.42-1.35)	0.344
< 10.0	1.30 (0.73-2.31)	0.373	88.1	< 0.001		
Adjusted educational						
Yes	1.00 (0.73-1.36)	0.997	42.1	0.189	0.89 (0.59-1.35)	0.590
No	1.12 (0.85-1.46)	0.414	80.6	< 0.001		
Adjusted BMI						
Yes	1.19 (0.68-2.08)	0.544	88.9	< 0.001	1.19 (0.67-2.11)	0.552
No	1.00 (0.88-1.13)	0.949	14.5	0.321		
Adjusted alcohol						
Yes	1.14 (0.78-1.66)	0.494	85.6	< 0.001	1.13 (0.75-1.70)	0.564
No	1.01 (0.86-1.19)	0.900	28.4	0.242		
Adjusted smoking						
Yes	1.13 (0.85-1.50)	0.409	84.4	< 0.001	1.05 (0.74-1.48)	0.798
No	1.08 (0.88-1.31)	0.465	0.0	0.398		
Adjusted PA						
Yes	1.28 (0.65-2.52)	0.477	94.7	< 0.001	1.28 (0.64-2.55)	0.483
No	1.00 (0.88-1.13)	0.960	12.6	0.333		

Table S3. Subgroup analysis for high versus low salted fish intake and the risk of gastric cancer

Group	RR and 95%CI	P value	Heterogeneity (%)	P value for heterogeneity	Ratio between subgroups	P value for interaction test
Country						
US or Europe	1.21 (0.80-1.83)	0.366	26.6	0.256	1.08 (0.68-1.72)	0.744
Asia	1.12 (0.91-1.39)	0.281	59.1	0.017		
Gender						
Men	1.14 (0.86-1.50)	0.357	28.6	0.221	1.28 (0.84-1.95)	0.248
Women	0.89 (0.65-1.22)	0.475	0.0	0.641		
Outcomes						
GC incidence	1.24 (1.04-1.48)	0.014	15.9	0.311	1.14 (0.81-1.59)	0.451
GC mortality	1.09 (0.82-1.45)	0.552	51.1	0.085		
Follow-up duration (years)						
≥ 10.0	1.00 (0.86-1.17)	0.994	0.0	0.467	0.79 (0.56-1.11)	0.167
< 10.0	1.27 (0.94-1.72)	0.120	51.7	0.082		
Adjusted educational						
Yes	1.00 (0.59-1.68)	1.000	-	-	0.87 (0.50-1.52)	0.623
No	1.15 (0.95-1.40)	0.154	54.0	0.021		
Adjusted BMI						
Yes	1.12 (0.80-1.56)	0.508	78.7	0.009	1.03 (0.70-1.52)	0.891
No	1.09 (0.89-1.33)	0.389	13.0	0.328		
Adjusted alcohol						
Yes	1.10 (0.83-1.46)	0.495	70.1	0.018	0.96 (0.66-1.39)	0.816
No	1.15 (0.90-1.47)	0.275	25.1	0.237		
Adjusted smoking						
Yes	1.18 (0.96-1.45)	0.122	49.8	0.052	1.24 (0.92-1.67)	0.152
No	0.95 (0.77-1.18)	0.633	0.0	0.493		
Adjusted PA						
Yes	1.47 (1.23-1.75)	< 0.001	-	-	1.46 (1.17-1.81)	0.001
No	1.01 (0.89-1.16)	0.840	0.7	0.431		

Table S4. Subgroup analysis for moderate versus low salted fish intake and the risk of gastric cancer

Group	RR and 95%CI	P value	Heterogeneity (%)	P value for heterogeneity	Ratio between subgroups	P value for interaction test
Country						
US or Europe	0.97 (0.67-1.42)	0.880	0.0	0.895	0.85	0.503
Asia	1.14 (0.85-1.51)	0.387	80.6	< 0.001	(0.53-1.37)	
Gender						
Men	0.99 (0.78-1.24)	0.901	0.0	0.508	1.52	0.035
Women	0.65 (0.48-0.90)	0.008	0.0	0.990	(1.03-2.25)	
Outcomes						
GC incidence	1.24 (0.93-1.65)	0.136	60.8	0.054	1.35	0.091
GC mortality	0.92 (0.76-1.12)	0.402	17.0	0.306	(0.95-1.91)	
Follow-up duration (years)						
≥ 10.0	0.89 (0.76-1.04)	0.138	0.0	0.478	0.66	0.004
< 10.0	1.35 (1.06-1.71)	0.014	36.6	0.192	(0.50-0.88)	
Adjusted educational						
Yes	-	-	-	-	-	-
No	1.10 (0.87-1.40)	0.436	73.7	<0.001		
Adjusted BMI						
Yes	1.22 (0.90-1.67)	0.202	75.6	0.017	1.34	0.112
No	0.91 (0.75-1.09)	0.313	6.6	0.369	(0.93-1.92)	
Adjusted alcohol						
Yes	1.22 (0.90-1.67)	0.202	75.6	0.017	1.34	0.112
No	0.91 (0.75-1.09)	0.313	6.6	0.369	(0.93-1.92)	
Adjusted smoking						
Yes	1.18 (0.95-1.47)	0.137	53.6	0.056	1.28	0.339
No	0.92 (0.58-1.46)	0.735	47.1	0.169	(0.77-2.14)	
Adjusted PA						
Yes	1.57 (1.32-1.86)	< 0.001	-	-	1.67	< 0.001
No	0.94 (0.82-1.08)	0.386	0.0	0.481	(1.34-2.08)	

Table S5. Subgroup analysis for high versus low processed meat intake and the risk of gastric cancer

Group	RR and 95%CI	P value	Heterogeneity (%)	P value for heterogeneity	Ratio between subgroups	P value for interaction test
Country						
US or Europe	1.20 (1.01-1.42)	0.035	60.4	0.027	0.71	0.598
Asia	1.70 (0.47-6.12)	0.414	82.3	0.018	(0.19-2.58)	
Gender						
Men	1.25 (0.82-1.90)	0.298	53.6	0.091	1.01	0.974
Women	1.24 (0.97-1.59)	0.088	21.3	0.283	(0.62-1.64)	
Outcomes						
GC incidence	1.26 (0.99-1.61)	0.062	67.8	0.014	0.95	0.863
GC mortality	1.33 (0.76-2.34)	0.315	69.9	0.036	(0.51-1.75)	
Follow-up duration (years)						
≥ 10.0	1.09 (0.92-1.27)	0.317	41.2	0.147	0.67	0.052
< 10.0	1.63 (1.12-2.36)	0.010	53.8	0.115	(0.45-1.00)	
Adjusted educational						
Yes	1.20 (1.01-1.42)	0.035	60.4	0.027	0.71	0.598
No	1.70 (0.47-6.12)	0.414	82.3	0.018	(0.19-2.58)	
Adjusted BMI						
Yes	1.21 (0.97-1.51)	0.092	71.4	0.015	0.93	0.754
No	1.30 (0.88-1.92)	0.184	55.7	0.080	(0.59-1.46)	
Adjusted alcohol						
Yes	1.25 (0.91-1.73)	0.173	71.7	0.014	0.99	0.971
No	1.26 (0.94-1.68)	0.117	62.6	0.045	(0.64-1.53)	
Adjusted smoking						
Yes	1.18 (0.98-1.42)	0.072	60.2	0.020	0.71	0.117
No	1.66 (1.13-2.44)	0.010	-	-	(0.46-1.09)	
Adjusted PA						
Yes	1.21 (0.74-1.99)	0.452	81.7	0.019	0.95	0.840
No	1.28 (1.02-1.61)	0.035	56.1	0.044	(0.55-1.63)	

Table S6. Subgroup analysis for moderate versus low processed meat intake and the risk of gastric cancer

Group	RR and 95%CI	P value	Heterogeneity (%)	P value for heterogeneity	Ratio between subgroups	P value for interaction test
Country						
US or Europe	1.02 (0.91-1.13)	0.778	13.8	0.326	1.01	0.975
Asia	1.01 (0.55-1.87)	0.975	-	-	(0.54-1.88)	
Gender						
Men	1.02 (0.86-1.21)	0.804	0.0	0.777	0.93	0.667
Women	1.10 (0.82-1.49)	0.519	33.8	0.209	(0.66-1.31)	
Outcomes						
GC incidence	1.04 (0.87-1.24)	0.645	35.4	0.200	1.03	0.798
GC mortality	1.01 (0.88-1.16)	0.888	0.0	1.000	(0.82-1.29)	
Follow-up duration (years)						
≥ 10.0	1.00 (0.88-1.13)	0.995	21.6	0.281	0.90	0.452
< 10.0	1.11 (0.87-1.41)	0.394	0.0	0.742	(0.69-1.18)	
Adjusted educational						
Yes	1.02 (0.91-1.13)	0.778	13.8	0.326	1.01	0.975
No	1.01 (0.55-1.87)	0.975	-	-	(0.54-1.88)	
Adjusted BMI						
Yes	1.03 (0.91-1.17)	0.623	31.0	0.226	1.11	0.587
No	0.93 (0.66-1.32)	0.701	0.0	0.762	(0.77-1.60)	
Adjusted alcohol						
Yes	1.04 (0.87-1.24)	0.645	35.4	0.200	1.03	0.798
No	1.01 (0.88-1.16)	0.888	0.0	1.000	(0.82-1.29)	
Adjusted smoking						
Yes	0.99 (0.90-1.09)	0.864	0.0	0.792	0.68	0.085
No	1.46 (0.95-2.25)	0.085	-	-	(0.44-1.05)	
Adjusted PA						
Yes	1.00 (0.84-1.18)	0.982	29.2	0.235	0.97	0.784
No	1.03 (0.91-1.17)	0.627	0.0	0.395	(0.79-1.20)	

Table S7. Subgroup analysis for high versus low miso-soup intake and the risk of gastric cancer

Group	RR and 95%CI	P value	Heterogeneity (%)	P value for heterogeneity	Ratio between subgroups	P value for interaction test
Country						
US or Europe	1.06 (0.76-1.46)	0.735	1.2	0.314	1.03	0.878
Asia	1.03 (0.87-1.21)	0.757	50.2	0.061	(0.71-1.48)	
Gender						
Men	0.95 (0.63-1.42)	0.792	65.7	0.020	0.78	0.345
Women	1.22 (0.88-1.68)	0.227	0.0	0.727	(0.46-1.31)	
Outcomes						
GC incidence	1.03 (0.94-1.14)	0.521	0.0	0.887	1.36	0.473
GC mortality	0.76 (0.33-1.72)	0.511	81.6	0.004	(0.59-3.11)	
Follow-up duration (years)						
≥ 10.0	0.99 (0.77-1.27)	0.939	67.2	0.016	0.93	0.649
< 10.0	1.06 (0.91-1.24)	0.440	0.0	0.842	(0.70-1.25)	
Adjusted educational						
Yes	1.02 (0.90-1.16)	0.791	0.0	0.583	1.02	0.889
No	1.00 (0.78-1.28)	0.996	56.8	0.041	(0.77-1.35)	
Adjusted BMI						
Yes	1.08 (0.91-1.28)	0.377	-	-	1.07	0.603
No	1.01 (0.84-1.22)	0.932	45.8	0.074	(0.83-1.38)	
Adjusted alcohol						
Yes	1.10 (0.94-1.28)	0.247	0.0	0.639	1.13	0.349
No	0.97 (0.79-1.21)	0.806	51.6	0.054	(0.87-1.48)	
Adjusted smoking						
Yes	1.00 (0.81-1.22)	0.967	55.2	0.048	0.88	0.385
No	1.13 (0.94-1.36)	0.204	0.0	0.565	(0.67-1.17)	
Adjusted PA						
Yes	1.08 (0.91-1.28)	0.377	-	-	1.07	0.603
No	1.01 (0.84-1.22)	0.932	45.8	0.074	(0.83-1.38)	

Table S8. Subgroup analysis for moderate versus low miso-soup intake and the risk of gastric cancer

Group	RR and 95%CI	P value	Heterogeneity (%)	P value for heterogeneity	Ratio between subgroups	P value for interaction test
Country						
US or Europe	0.92 (0.58-1.45)	0.718	-	-	0.89	0.635
Asia	1.03 (0.94-1.12)	0.542	0.0	0.990	(0.56-1.42)	
Gender						
Men	0.95 (0.80-1.12)	0.536	0.0	0.956	0.79	0.110
Women	1.20 (0.95-1.51)	0.132	0.0	0.861	(0.59-1.05)	
Outcomes						
GC incidence	1.02 (0.92-1.13)	0.702	0.0	0.965	0.99	0.914
GC mortality	1.03 (0.89-1.19)	0.700	0.0	0.676	(0.83-1.18)	
Follow-up duration (years)						
≥ 10.0	1.02 (0.92-1.13)	0.667	0.0	0.958	1.00	1.000
< 10.0	1.02 (0.88-1.18)	0.752	0.0	0.878	(0.84-1.20)	
Adjusted educational						
Yes	1.00 (0.88-1.14)	0.976	0.0	0.699	0.96	0.646
No	1.04 (0.93-1.15)	0.509	0.0	0.977	(0.81-1.14)	
Adjusted BMI						
Yes	1.06 (0.90-1.25)	0.497	-	-	1.05	0.617
No	1.01 (0.92-1.11)	0.818	0.0	0.991	(0.87-1.27)	
Adjusted alcohol						
Yes	1.06 (0.90-1.25)	0.497	-	-	1.05	0.617
No	1.01 (0.92-1.11)	0.818	0.0	0.991	(0.87-1.27)	
Adjusted smoking						
Yes	1.02 (0.92-1.13)	0.711	0.0	0.908	0.99	0.914
No	1.03 (0.89-1.19)	0.688	0.0	0.902	(0.83-1.18)	
Adjusted PA						
Yes	1.06 (0.90-1.25)	0.497	-	-	1.05	0.617
No	1.01 (0.92-1.11)	0.818	0.0	0.991	(0.87-1.27)	
