# Supplementary material for: Dietary Salt Intake and Gastric Cancer Risk: A Systematic Review and Meta-Analysis
Source: Front Nutr. 2021 Dec 8;8:801228. doi: 10.3389/fnut.2021.801228 (PMC8692376; doi:10.3389/fnut.2021.801228)
Supplement: Supplementary file 3 [file Presentation_3.pdf]

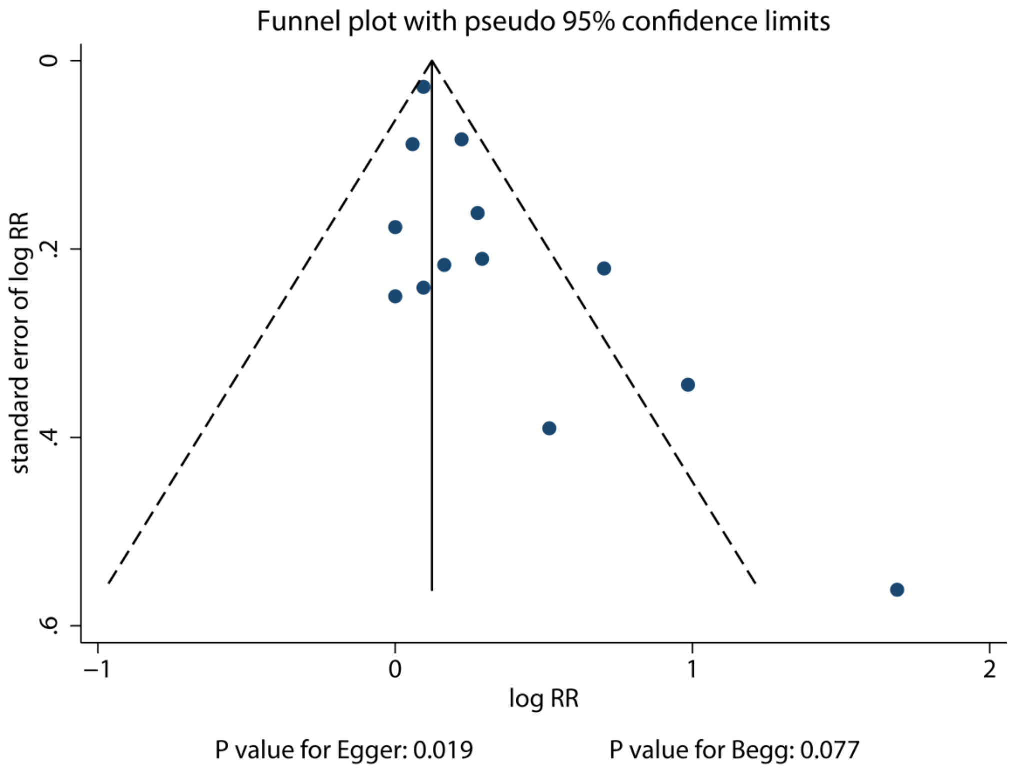

Figure S1. Funnel plot for high versus low salt intake and the risk of gastric cancer

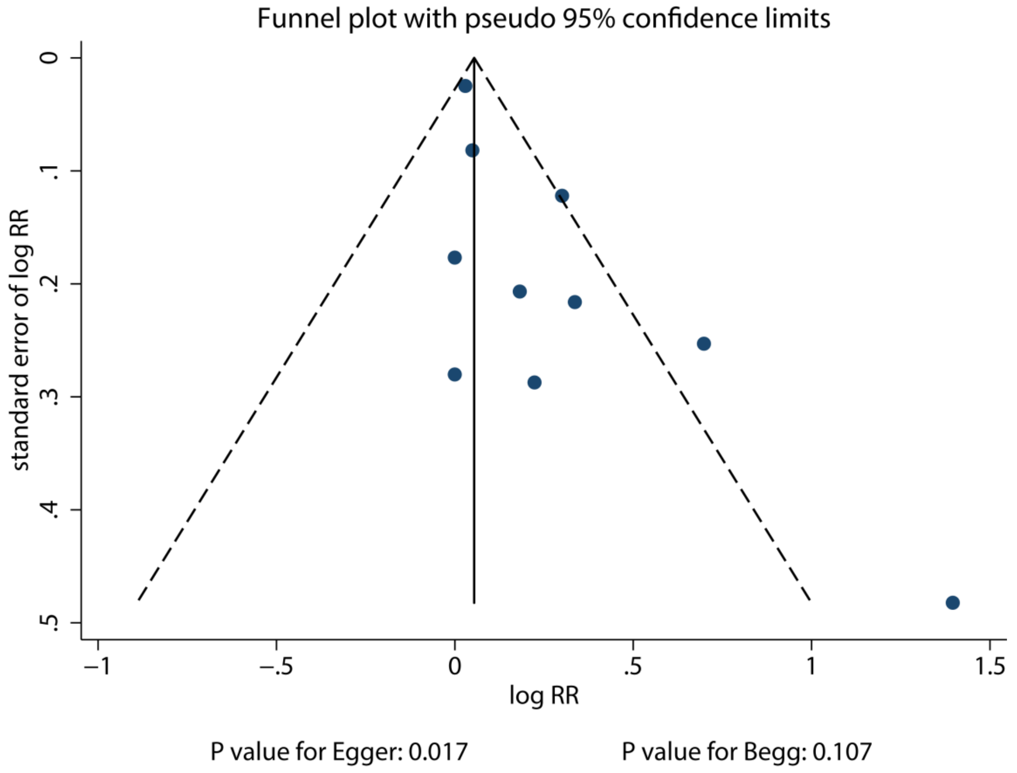

Figure S2. Funnel plot for moderate versus low salt intake and the risk of gastric cancer

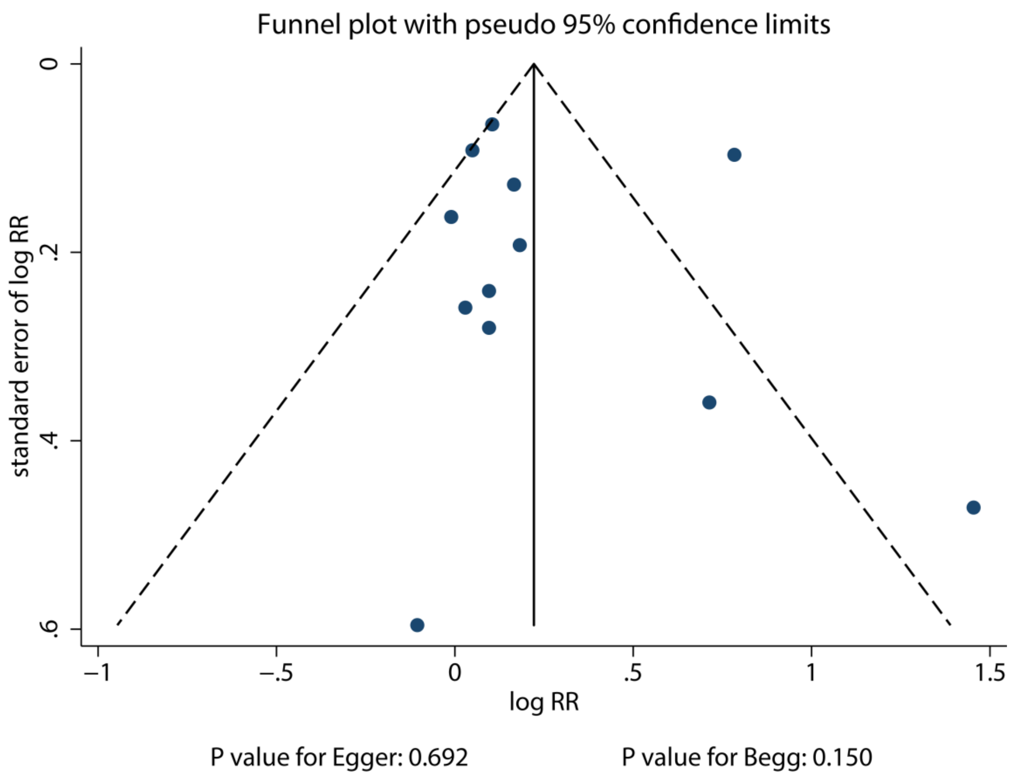

Figure S3. Funnel plot for high versus low pickled food intake and the risk of gastric cancer

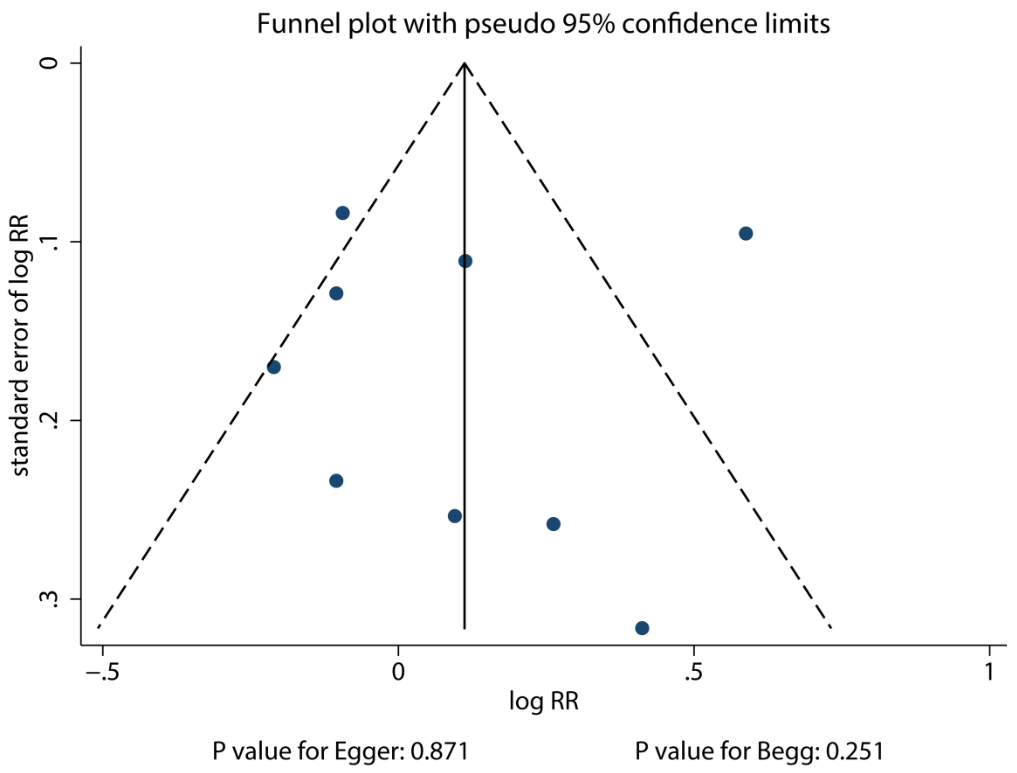

Figure S4. Funnel plot for moderate versus low pickled food intake and the risk of gastric cancer

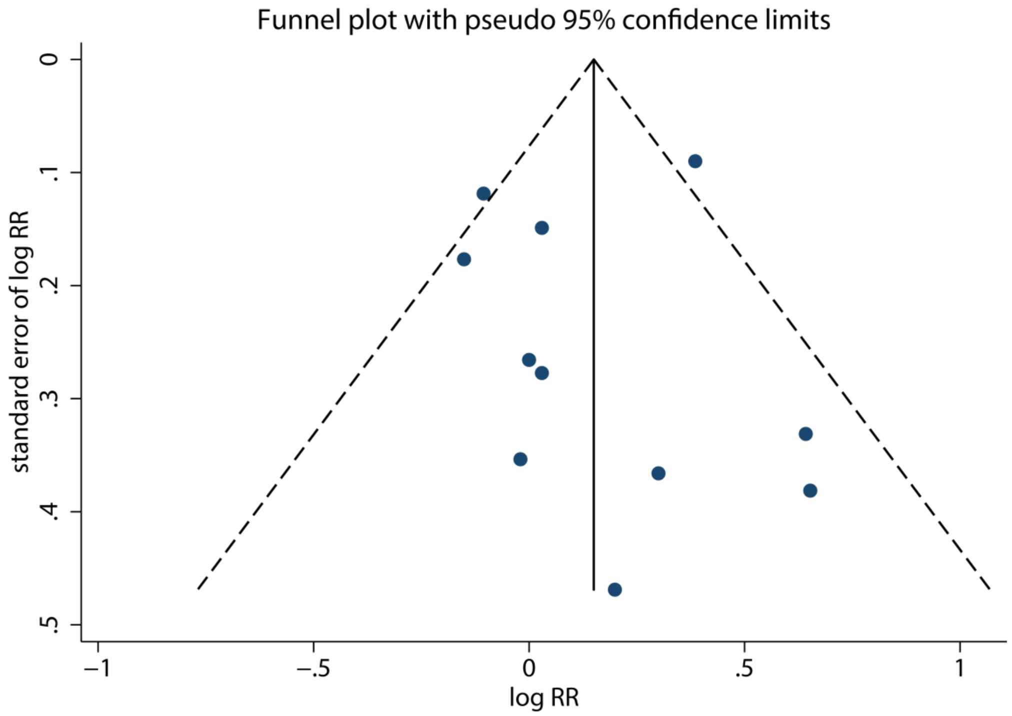

P value for Egger: 0.929 P value for Begg: 0.161

Figure S5. Funnel plot for high versus low salted fish intake and the risk of gastric cancer

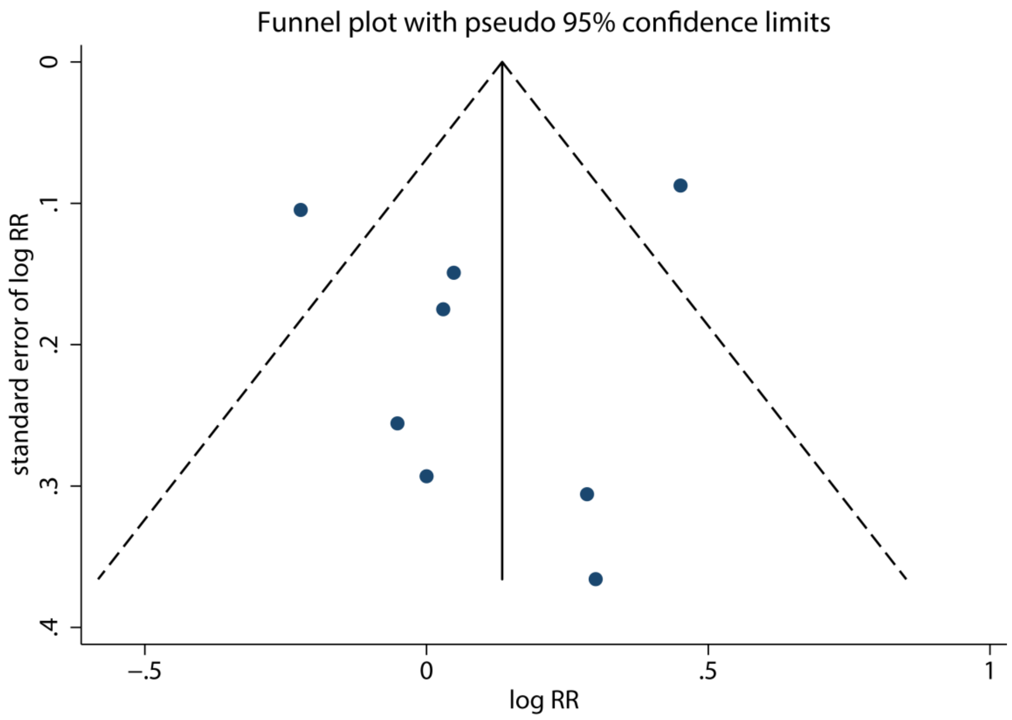

P value for Egger: 0.669 P value for Begg: 0.536

Figure S6. Funnel plot for moderate versus low salted fish intake and the risk of gastric cancer

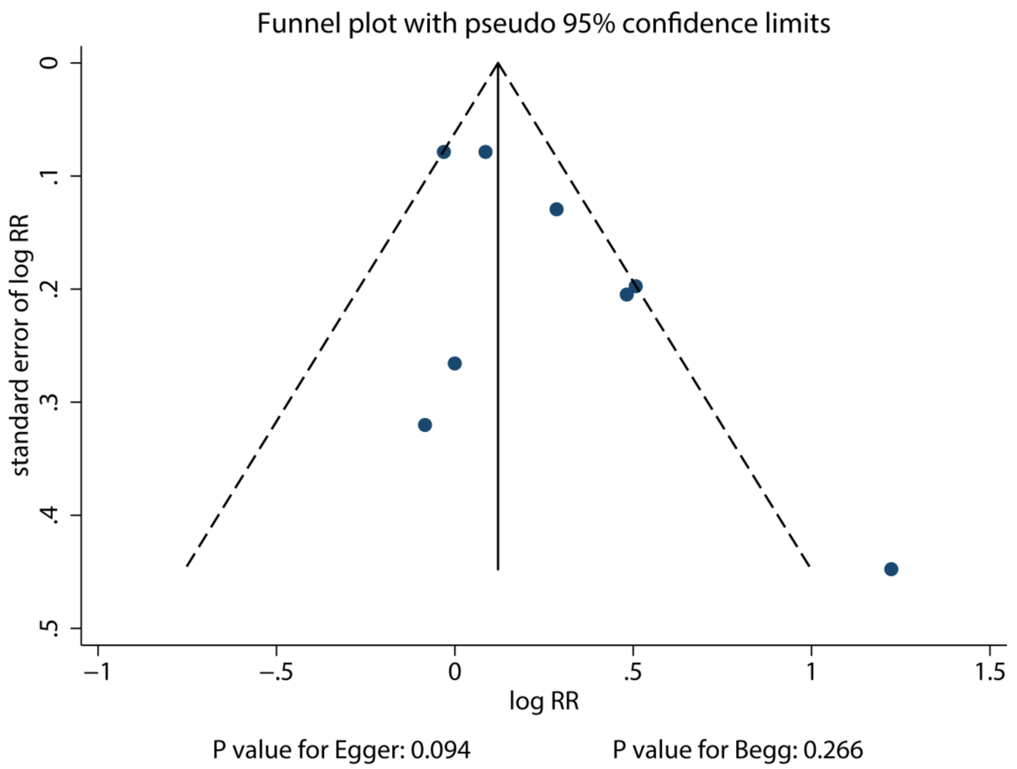

Figure S7. Funnel plot for high versus low processed meat intake and the risk of gastric cancer

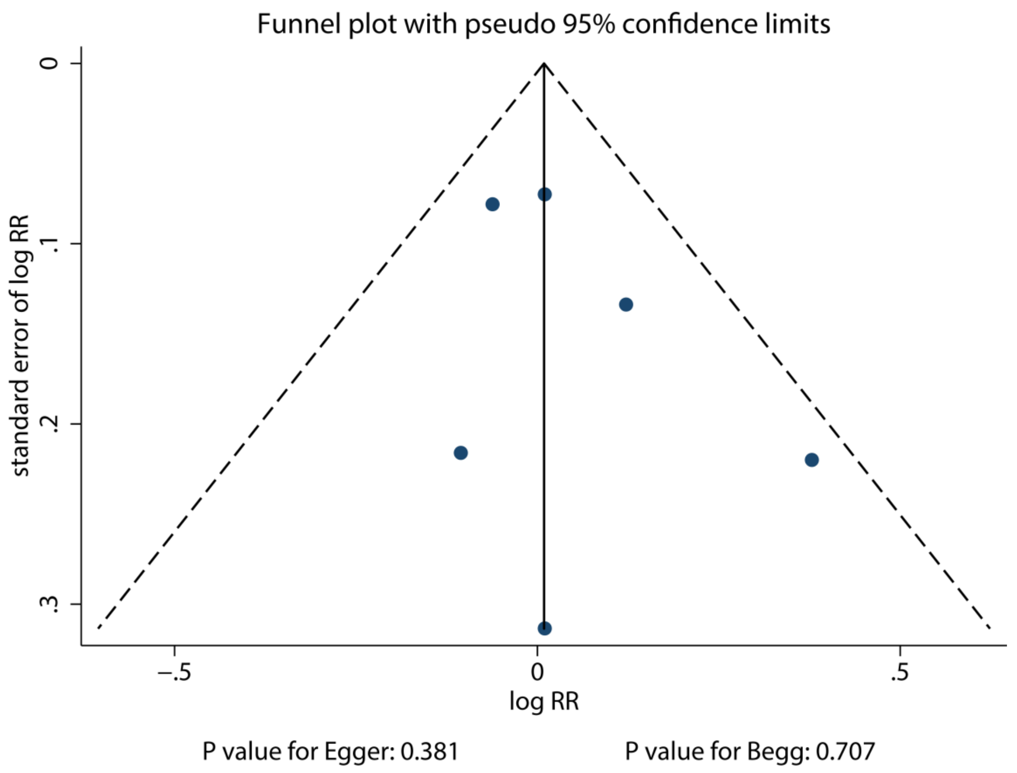

Figure S8. Funnel plot for moderate versus low processed meat intake and the risk of gastric cancer

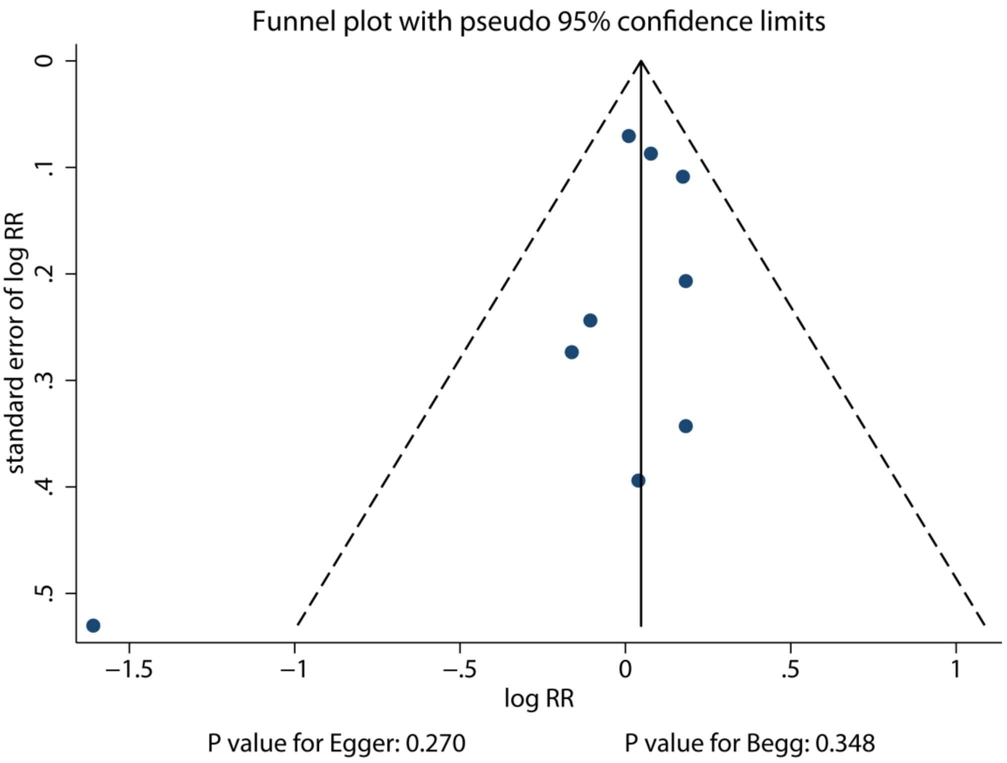

Figure S9. Funnel plot for high versus low miso-soup intake and the risk of gastric cancer

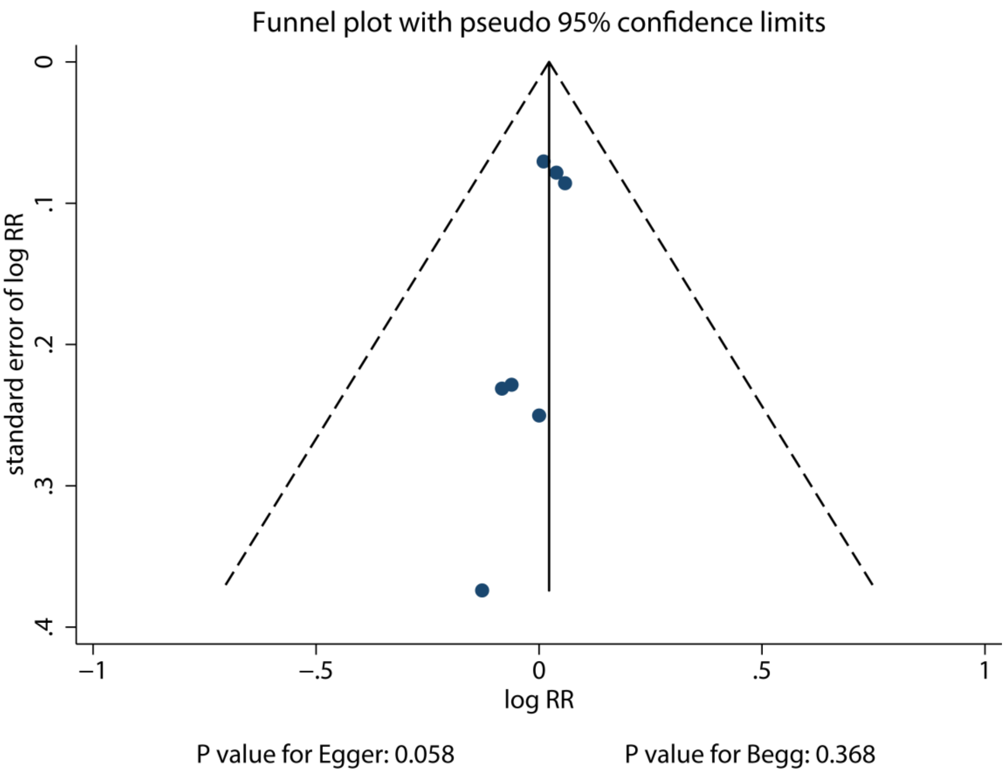

Figure S10. Funnel plot for moderate versus low miso-soup intake and the risk of gastric cancer
